# Cavity QED in a high NA resonator

**DOI:** 10.1126/sciadv.ads8171

**Published:** 2025-02-26

**Authors:** Danial Shadmany, Aishwarya Kumar, Anna Soper, Lukas Palm, Chuan Yin, Henry Ando, Bowen Li, Lavanya Taneja, Matt Jaffe, Schuster David, Jon Simon

**Affiliations:** ^1^ Department of Physics, Stanford University, Stanford, CA, USA.; ^2^ Department of Physics and Astronomy, Stony Brook University, Stony Brook, NY, USA.; ^3^ Department of Applied Physics, Stanford University, Stanford, CA, USA.; ^4^ Department of Physics, The University of Chicago and the James Franck Institute, Chicago, IL, USA.; ^5^ Department of Physics, Montana State University, Bozeman, MT, USA.

## Abstract

From fundamental studies of light-matter interaction to applications in quantum networking and sensing, cavity quantum electrodynamics (QED) provides a toolbox to control interactions between atoms and photons. The coherence of interactions is determined by the single-pass atomic absorption and number of photon round-trips. Reducing the cavity loss has enabled resonators supporting 1 million roundtrips but with limited material choices and increased alignment sensitivity. Here, we present a high–numerical aperture, lens-based resonator that pushes the single-atom single-photon absorption probability near its fundamental limit, reducing the mode size at the atom to order λ. This resonator provides a single-atom cooperativity of 1.6 in a cavity where the light circulates ∼10 times. We load single ^87^Rb atoms into this cavity, observe strong coupling, and demonstrate cavity-enhanced atom detection with fidelity of 99.55(6)% and survival of 99.89(4)% in 130 μs. Introducing intracavity imaging systems will enable cavity arrays compatible with Rydberg atom array computing technologies, expanding the applicability of the cavity QED toolbox.

## Teaser

By engineering a multiple-pass standing wave of light <1 μm size at the center, single atoms can be trapped and read out fast.

## INTRODUCTION

Cavity quantum electrodynamics (QED), the study of light-matter interaction through coupling of emitters to the field of an optical resonator, enables coherent information exchange between material and photonic degrees of freedom, with wide-ranging applications from qubit state detection (*1*–*4*), to sensing (*5*–*8*), and networking (*9*–*11*). Such tools apply not only to coupling of light to laser-cooled atoms and ions (*12*–*15*) but also transmon qubits, rare earth ionic dopants (*16*, *17*), color centers (*18*–*20*), quantum dots (*21*, *22*), and other optically active emitters.

The fundamental figure of merit that controls the coherence of light-matter interactions is the cooperativity, given by $C=\frac{4g^{2}}{\kappa \Gamma }$, where *g* is the coherent information exchange rate, κ is the full width at half intensity maximum of the resonator, and Γ is the decay rate of the material excitation (*23*). For applications in quantum information science, the number of coherent information exchanges is approximately $\sqrt{C}$ (section S5.2), and the infidelity of a cavity mediated gate is $\epsilon \approx \frac{2\pi }{\sqrt{C}}$ [(*24*, *25*) and section S5.2]. Cavities can also be harnessed for Purcell enhanced atomic state detection, where the ratio of atomic emission into the cavity versus free space is *C* (*23*); equivalently, the Purcell enhanced collection solid angle is 4π × *C*.

For a closed optical transition such as those available in alkali atoms, the cooperativity of a macroscopic resonator may be expressed in terms of the resonator geometry $C=\frac{12}{\pi^{2}}\frac{F}{2\pi }\frac{\lambda^{2}}{w^{2}}$ [(*23*) and section S5.20], where *F* is the resonator finesse defined so that *F*/(2π) is the mean number of times the light passes the atom within the cavity, *w* is the optical mode size at the atom, and λ is the wavelength of the optical transition. At fixed λ, this expression suggests two routes to strong coupling (*C* > 1 rather than *g* > γ, κ)—large finesse or small mode waist.

Finesses above 10^5^ have been achieved using ion-beam sputtered dielectric coatings on super polished (*26*) or laser ablated (*27*) substrates, and recently, finesses above 10^6^ have been achieved using reactive ion etching (*28*) to reduce surface roughness. Further gains would necessitate breakthroughs in atom-scale surface polishing and part-per-million-level coating absorption.

The decades-long effort to reduce the mode size *w* has spanned numerous approaches: In two-mirror cavities, the near-confocal geometry (*29*) with length *L* equal to the mirror radius of curvature *R* is a compromise between resonator alignment sensitivity and optical access that results in a mode waist of $w=\sqrt{\frac{\mathrm{R\lambda}}{2\pi }}$. Efforts to reduce the mode waist in two-mirror cavities beyond this limit have focused on either (i) small mirror separation *L* ≪ *R* (near-planar resonators), providing a substantial reduction in mode waist (*12*, *27*) at the expense of reduced optical access and sensitivity to E-fields from proximal surfaces; or (ii) near-concentric resonator geometry *L* ≈ 2*R*, providing a few-fold waist reduction compared to the confocal geometry at the expense of increased alignment sensitivity (*4*, *30*, *31*).

Achieving yet-smaller mode waists requires exploring more sophisticated resonator geometries. For example, by using an additional long propagation arm and/or a pair of convex mirrors, the bowtie resonator allows for mode waists down to a limit imposed by astigmatism induced by off-axis incidence on the focusing mirrors (*32*, *33*). Such resonators have demonstrated waists down to ∼7 μm (*34*), the limit thus far achieved for high-finesse macroscopic Fabry-Pérot cavities. Wavelength-scale resolutions have been observed in a multipass imaging system for biological samples (*35*) but never in a macroscopic device compatible with atomic cavity QED. Nanoscale and fiber cavities have been used to achieve finer resolutions but have reduced optical access, presenting a challenge for integration with fast single atom control and Rydberg states due to surface fields (*36*).

In this work, we present a macroscopic resonator with a λ-scale mode waist. This enables us to enter the strong coupling regime of cavity QED in a resonator with finesse below 50. This small waist also opens the door to integration with state-of-the-art neutral atom arrays by allowing site-selective readout of single atoms in tightly (5 μm) spaced arrays, a challenge for near-concentric cavities with 15- to 20-μm waists (*4*, *30*, *31*). In Results, we introduce this resonator geometry and describe its construction and stability, going on to describe the process of loading/detecting a single atom and use it to characterize the resonator with a measurement of the atom-cavity coupling. In Discussion, we summarize fast atom detection and conclude by describing opportunities to leverage this approach for emerging quantum science and technology.

## RESULTS

**Fig. 1.**
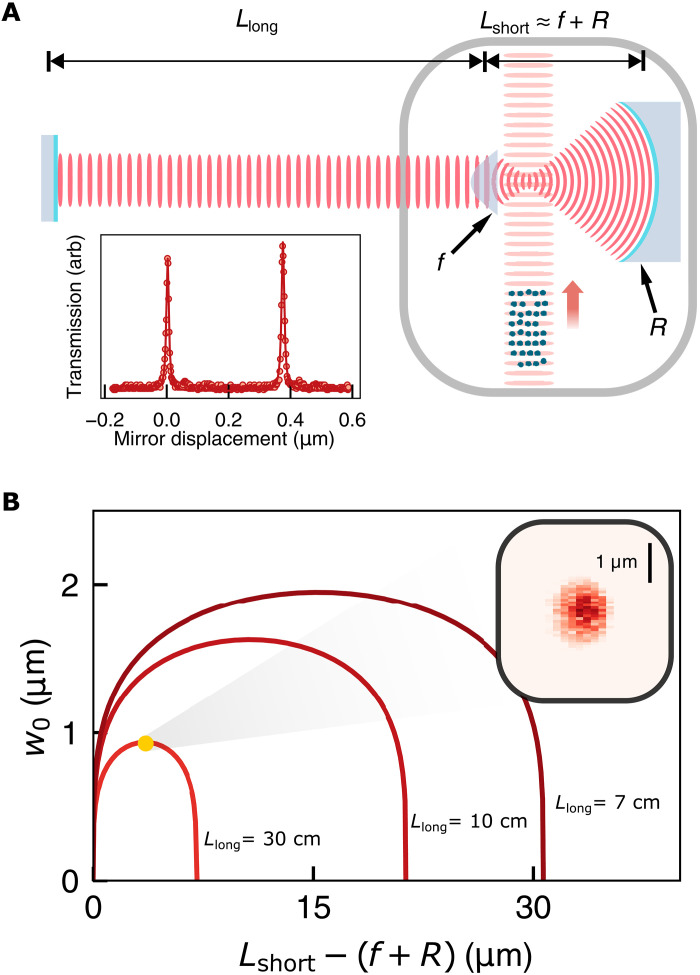
Resonator design. The heart of the apparatus, shown in (**A**), is a cavity consisting of a spherical mirror (radius *R*) that focuses light down to a submicron spot, a high-NA aspheric lens (focal length *f*) that recollimates it, and lastly a long propagation (length *L*_long_). This long propagation distance necessitates that the vacuum chamber window (gray) resides inside of the resonator. An optical conveyor belt vertically transports ^87^Rb atoms to the resonator from a magneto-optical trap. See fig. S2 for a detailed schematic of the optics setup. Inset: A cavity transmission spectrum (arbitrary scale) reveals a fitted finesse *F* = 40 (2); for a 480-MHz FSR, the linewidth is thus κ = 2π × 12.4(7) MHz. arb, arbitrary units. (**B**) The paraxially computed mode waist (*w*_0_) is plotted versus the distance between the aspheric lens and spherical mirror, quantified as the deviation from overlapping lens and mirror foci. Introduction of the asphere within the cavity allows for the exploration of resonators that are asymmetric, consisting of two propagation arms of unequal lengths. For *L*_long_ ≫ *L*_short_, the width of the stability region (*f*^2^/*L*_long_) compresses, leading in the extreme case (*L*_long_ ≈ 30 cm) to a submicron waist that persists even in the middle of the stable range, rather than exclusively near the (highly-sensitive) edges as is common in macroscopic two mirror cavities. Inset: A direct measurement of the mode waist (via scanning a nanopore across the mode; see section S5.16) bounds the mode size at a maximum 1/*e*^2^ radius of 1000 nm.

The principal innovation in our resonator design is the integration of a high–numerical aperture (NA) aspheric lens (focal length *f*) within a two mirror Fabry-Perot cavity. This lens divides the resonator into a short arm (length *L*_short_) between the lens and a spherical mirror (radius of curvature *R*) and a long arm (length *L*_long_) between the lens and a planar mirror (see Fig. 1A). When *L*_long_, *R* ≫ *f*, a small waist appears in the short arm near the center of curvature of the spherical mirror and focus of the aspheric lens. The size of this waist, $w_{0}\approx \sqrt{\frac{f}{L_{\text{long}}}}\times \sqrt{\frac{\mathrm{f\lambda}}{2\pi }}$, is very similar to that of a confocal cavity but with additional demagnification induced by the long arm. The length of the long propagation arm *L*_long_ must be equal to the Rayleigh range of the waist at the planar end mirror to ensure that the spatial phase profile of the cavity mode matches the shape of the optics. Intuitively, this provides a relation between the length of the long arm and the waist size at the atoms: The arm needs to be long enough to support a cavity modes that fills the *NA* of the asphere.

There are several important features of note: (i) If the long arm is long enough, submicron waists are possible at the center of the stability diagram, limited by clipping and aberration due to finite lens NA (see section S5.17); (ii) using an asphere allows us to minimize aberrations while maintaining a high NA, unlike a bowtie resonator with spherical mirrors where astigmatism limits the achievable waist (*32*); (iii) with a round trip Gouy phase of π/2, the transverse mode structure of the cavity is neither that of a concentric or a confocal resonator, suppressing mode mixing due to lower order aberrations (*37*). For our parameters (*L*_long_ = 30 cm, *f* = 1.45 mm), we expect a waist of 930 nm (see Fig. 1B and section S5.9).

In our implementation of the resonator geometry described above, we mount the asphere and the curved mirror in an ultra-high vacuum load-lock system [see fig. S9 and (*38*)]. The mount is a custom designed three-axis piezo-driven and mechanically multiplied flexure stage, providing precise control of the relative position of the asphere and mirror, crucial to aligning the cavity in the presence of drifts (see section S5.18). We place the in-coupling (*R* = 98%) flat end mirror of the cavity outside vacuum and use it primarily to in-couple our probe and trapping light. We insert a pellicle right after the flat mirror to pick-off a controllable (angle-dependent) fraction of the light circulating in the cavity to either measure cavity transmission or atomic fluorescence. Furthermore, we use an electro-optic modulator between the pellicle and vacuum window for high bandwidth cavity locking. We directly verify a micron-scale waist in an out-of-vacuum test setup by using a gold coated film containing a 200-nm diameter aperture (see section S5.16).

To conclusively demonstrate high cooperativity and the utility of the platform in atom-based quantum information protocols, we trap a single atom at the waist of the resonator and use it to probe the cavity. Our experiments begin with a cloud of laser cooled ^87^Rb atoms from a magneto optical trap which is transported to the small cavity waist location in a one-dimensional optical conveyor belt (see Fig. 1A). Additional polarization gradient cooling (PGC) at the cavity waist location loads the atoms into an intracavity dipole trap at 785 nm with a peak depth of *U*_0_/*k*_B_ = 2.0 (5) mK (measured as half the trap-induced shift of the atom-cavity resonance condition).

It is essential that atoms within the cavity be able to collide to ensure that the PGC process drives parity projection (*39*). As such, the atoms are first loaded into a dipole trap rather than a lattice. We wash out the cavity standing wave by applying sidebands to the 785 nm cavity lattice laser, to excite cavity modes 10 free spectral ranges (FSRs) away, creating a dipole trap at the small waist location (see section S5.4). Once a single atom is loaded into the dipole trap, we remove the sidebands while cooling over 2 ms, reloading the atom into a single cavity lattice well. The trapping wavelength must thus be chosen to align the 780- and 785-nm cavity standing waves at the location of the small cavity waist (see section S5.5). To probe the system, we monitor the PGC-induced fluorescence scattered into the cavity, picked off by the pellicle, and directed into a pair of single photon counting modules (SPCMs).

**Fig. 2.**
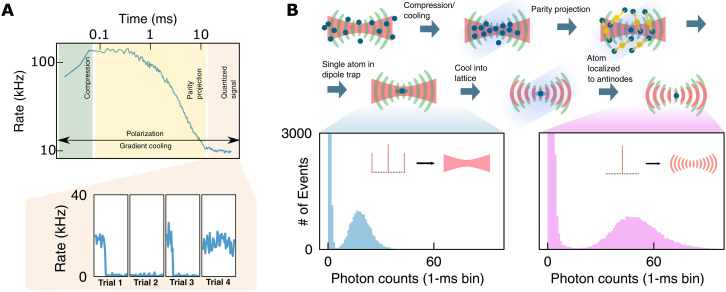
Single atom loading. **(A)** Early time dynamics of the Rb cloud cavity emission, measured as the rate of detected photons on an SPCM. First (dark green), the signal increases as atoms are cooled into the cavity-enhanced trap. After reaching a maximum (yellow), the atoms begin to parity project with timescale set by near-resonant photo-association as described in (*32*), below our current SNR-limited resolution. Inset: Last (orange), the signal settles to quantized levels with time traces below showing the single atom signal in individual shots 30 to 190 ms after loading begins. (**B**) Full schematic of loading/localization schemes. First, atoms are released from the transport lattice and cooled into the trap (red) by the three-dimensional molasses beams (dark blue), where the intracavity dipole trap is created by adding off-resonant sidebands 10 FSRs away (see section S4.4). Near the small waist, atoms undergo light-assisted collisions until only 0 or 1 atom remains. In light blue, we show the histogram of scattering statistics with 1-ms bins for a single atom in a dipole trap. This corresponds to a scattering rate of ≈20 kHz. The single atom in the dipole trap samples nodes and antinodes of the cavity lattice (light green) equally, cutting the effective cooperativity by a factor of two. To improve, we alter the trapping light from a dipole trap to a lattice by removing the far sidebands which, with correct phase matching, localizes the atom to the readout antinode. We plot a histogram of statistics for the localized single atom with 1-ms bins (pink) showing a scattering rate of ≈50 kHz. Inset: Schematic of sideband scheme in dipole trap and lattice, with black dots indicating individual FSRs of cavity and tones indicated with red lines.

**Fig. 3.**
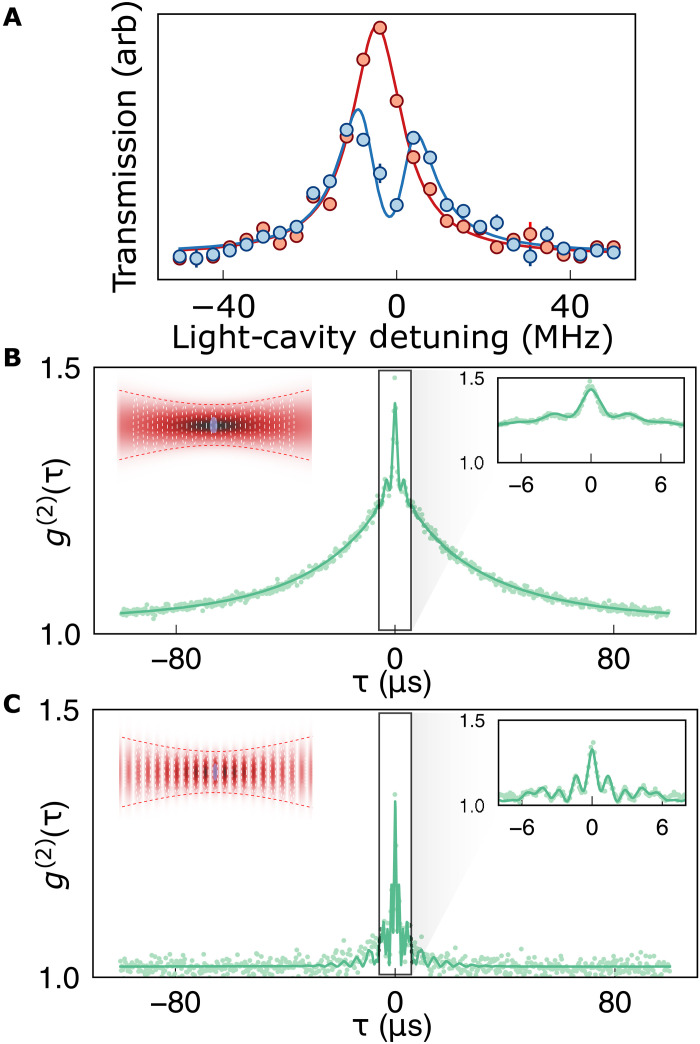
Trapped atom characterization. Once an atom is detected, we probe its cavity coupling. (**A**) Cavity transmission versus probe laser frequency, with/without atom in blue/red; the mode splitting apparent in the presence of the atom indicates strong light-matter coupling with *C* = 1.6(2). (**B**) Two-time correlator *g*^(2)^(τ) of cavity fluorescence from a dipole-trapped atom versus time difference τ. The fast (3 μs) oscillations (inset) come from radial oscillations away from the cavity axis where coupling is strongest, rapidly damped by PGC. The slow decay over ∼50 μs comes from overdamping the weak axial dipole trap. A fit (solid, see section S5.12) indicates that axial and radial temperatures differ by a factor ∼100 (*T*_*z*_ ≈ 5 μK, *T*_*x*,*y*_ ≈ 600 μK), while axial and radial PGC-damping rates differ by a factor ∼10 (γ_*x*,*y*_ = 500 ms^−1^, γ_*z*_ = 5000 ms^−1^). (**C**) *g*^(2)^(τ) of lattice trapped atom; the absence of slow decay indicates stronger axial trapping of the lattice. The fit (solid, see section S5.12) reveals equal axial and radial temperatures = 200 μK, with axial and radial damping rates differing by a factor of four (γ_*x*,*y*_ = 112 ms^−1^, γ_*z*_ = 400 ms^−1^). [(B) and (C)] Insets depict trapping potential in red, light-matter coupling in white, and extent of atomic motion in purple.

We first study the atom-cavity interaction with outcoupling *T* = 4% well below the internal cavity round trip loss *L*_rt_ = 11.7% to achieve a near-maximal finesse of F=40.0(1) and hence a predicted single-atom cooperativity *C*_max_ = 6. Figure 1A shows averaged fluorescence as an atomic ensemble is cooled into the intracavity dipole trap. An initial compression leads to an increase in the fluorescence, followed by a sharp decrease during parity projection, after which the signal settles to quantized levels (Fig. 1A). This quantized signal arises from discrete atomic occupancy of the cavity trap, dominated by either 0 or 1 atom loaded (*40*). Figure 2B shows the histogram of collected fluorescence photon numbers from dipole trapped atoms, with a clear separation between the background and a single atom loaded. After transferring to an intracavity lattice with a depth of 800 (80) μK, the signal is enhanced twofold because the atom is better localized to an antinode of the 780-nm cavity field (see section S5.5). Armed with the ability to condition measurements on the presence of an atom, we extract the single-atom cavity coupling strength by measuring the vacuum Rabi splitting. In Fig. 3A, we plot the cavity transmission as we scan a 780-nm probe laser across the cavity resonance, both with (blue) and without (red) an atom. A single-atom vacuum Rabi splitting is clearly visible with a fitted coupling strength *g* = 2π × 5.6(3) MHz and a slight asymmetry arising from an atom-cavity detuning of Δ = 1.5(6) MHz. Combined with the atomic linewidth Γ = 2π × 6.065 MHz and our measured κ = 2π × 13.3(1) MHz, this yields a cooperativity, *C* = 1.6(2), placing us in the regime where emission into the cavity is more likely than into free space. This cooperativity is about a factor of ∼4 lower than the maximum achievable due to a combination of cavity birefringence (≈2× reduction) and atomic motion (≈2× reduction) (see section S5.15). It is also possible to express these quantities as decay rates of fields and to fit accordingly (*41*). We also leverage our ability to perform atom-conditioned measurements to extract the second order correlation function, *g*^(2)^(τ) of the atomic fluorescence, taken both in the dipole trap (Fig. 3B) and lattice (Fig. 3C) (see section S5.8 for parameters). Both plots exhibit microsecond-scale oscillations indicative of radial trapping that reaches equilibrium with the optical molasses (see section S5.12 for derivation of the solid fits); this oscillation reflects the fact that atoms are more likely to scatter at times when they are near cavity axis [large *g*_2_(τ = 0)] and less likely to scatter a half trap period later when the atom has oscillated away from the cavity axis [small *g*_2_(τ = *T*_trap_/4)]. Data from the dipole-trapped atom exhibit an additional slow decay indicative of overdamping of the weakly trapped axial motion. By contrast, the lattice-trapped atom is strongly confined in the axial direction and so exhibits an additional, even faster (axial) oscillation in the *g*_2_ instead place of overdamped decay apparent for the dipole-trapped atom.

## DISCUSSION

The low outcoupling probability used for the aforementioned experiments is not optimal for atomic state detection in the presence of fixed internal cavity losses. The optimum outcoupling is a tradeoff: Increased outcoupling improves the fraction of cavity photons leak out of the cavity before they are lost to scattering/absorption of cavity optics, while reduced outcoupling improves the cooperativity and thus the probability that the atom scatters light into the cavity in the first place. As we show in section S5.1, the optimal operating point occurs when the outcoupling probability is $L_{\text{int}}\sqrt{1+C_{\text{max}}}$, where *C*_max_ is the cooperativity with no outcoupling and *L*_int_ is the internal cavity loss per round trip. To further demonstrate the flexibility of our platform, we enter this optimal regime by tuning the angle of the intracavity pellicle to outcouple more light (optimized at 20%, with an estimated new cooperativity of ≈0.8).

**Fig. 4.**
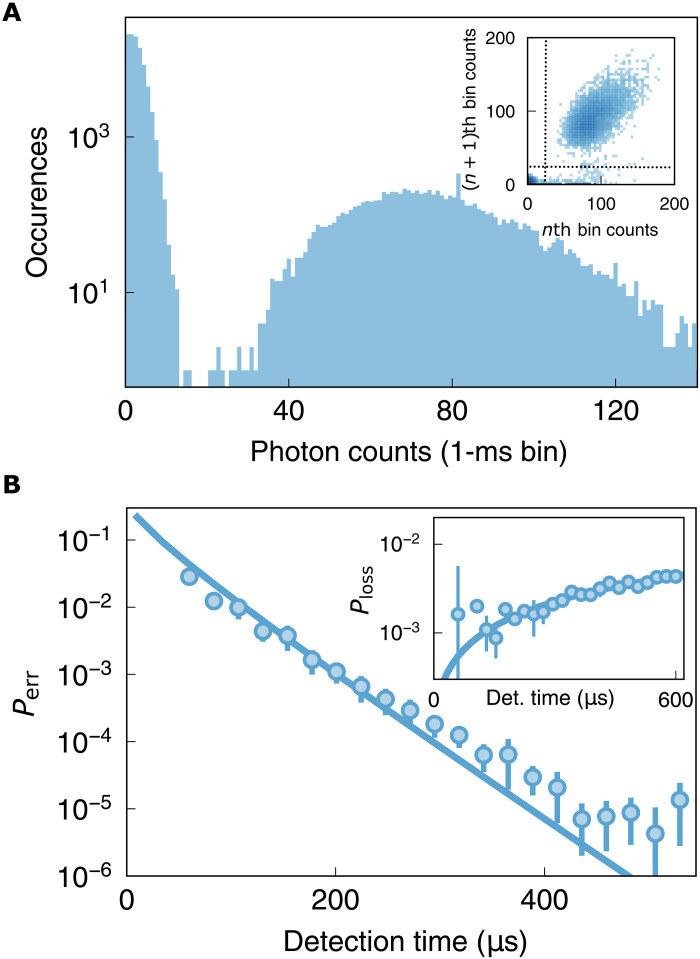
Characterizing atom detection fidelity. **(A)** Histogram of cavity fluorescence photons collected in 1-ms bins from a single lattice-trapped atom at larger *T* = 20% cavity outcoupling. The measured scattering rate is ∼75 kHz, 1.7× higher than that measured in the same configuration at *T* = 4% outcoupling. See section S5.1 for derivation of relationship between total cavity collection rate and outcoupling. Inset: Correlations between subsequent measurements of a single atom: Upper-right quartile reflects the persistence of an atom through adjacent measurements; lower-right quartile reflects an atom lost between the measurements; lower-left quantile reflects measurements both with no atom; upper-left quantile reflects appearance of an atom between measurements, indicating imperfect dispersal of the transported cloud. The elliptical shape of the data in the upper-right quadrant indicates correlations in scattering rate between subsequent measurements, likely due to site to site variation in cavity coupling strength. (**B**) The model-independent fidelity estimates are plotted versus measurement time, showing a fidelity of 99.55 (6)% reached at a measurement time of 130 μs. The inset depicts the corresponding estimated loss probability versus the imaging time and with a model (solid curve) for a survival rate of 99.89 (4)% and a 112 (8)–ms lifetime (see section S5.7).

Figure 4A shows atom detection for optimized outcoupling. Compared to the low-outcoupling configuration, we observe an increase in the detected photon rate by a factor of ∼1.7 (Fig. 4A), consistent with expectations (see section S5.1). A systematic, model-independent analysis of atom detection [see (*42*) and section S5.19] reveals that, within 130 μs, we are able to detect an atom with fidelity of 99.55(6)% (see Fig. 4B) while maintaining an atom survival probability of 99.89(4)% (see Fig. 4B, inset), consistent with the atom lifetime in the presence of the molasses light. By comparison, a simple molasses applied to an atom array over 80 ms enables 99.99% fidelity and survival (*43*),while more optimized, finite field pulsed molasses enables 99.8% fidelity in 500 μs (*44*). Comparing to the current integrations of readout of neutral atom arrays enhanced by near-concentric cavities, our resonator performs comparably with readout speeds of 30 μs achieved in those systems (*45*). It is important to note that the typical waist size (15 to 20 μm) of the near-concentric resonators exceeds the atom spacing in arrays. The readout numbers are limited by the internal losses of our resonator, without which we could increase the resonator finesse, and thereby the Purcell enhancement factor of the resonator while maintaining efficient resonator outcoupling. Reducing these losses, through a combination of better antireflection coatings and lower surface roughness, will immediately reduce the readout time. We postulate that our observed atom lifetime of 112(8) ms is limited by a combination of intensity noise of the trapping laser arising from a combination of cavity shaking and laser frequency noise, to be improved upon in upcoming work.

Here, we have introduced an approach to cavity QED that leverages high-NA, low-finesse cavities to enter the strong coupling regime. We load a single atom into such a cavity, with a finesse *F* = 40, harnessing cavity enhancement to create first a dipole trap and then a lattice, which efficiently confines the atom. We validate that we are in the strong coupling regime via an atom-conditioned vacuum Rabi splitting measurement, proving the presence of a single atom via a quantized signal. Temporal correlations of the light scattered by the atom into the cavity enable precise measurements of both atom temperature and PGC damping coefficients. When we optimize the cavity outcoupling to maximize total atomic light scattering, we are able to detect a single atom with a fidelity of 99.55(6)% in a time of 130 μs, with a survival rate of 99.9%.

The parameters of our platform are far from optimized: A nonbirefringent cavity will provide 2× the collection efficiency, while lower atom temperature will provide an additional factor of 1.8; free-space photon counters provide an extra factor 2 versus their fiber-coupled counterparts, for a projected detection time of 20 μs. Reduced intracavity loss will yield further improvements in collection efficiency and cooperativity, enabling nondestructive atom detection in less than ∼10 μs. See section S5.21. The minimal finesse requirements mean that it will be possible to strongly couple the atom to more sophisticated intracavity optics: Nonlinear crystals will enable in situ wavelength conversion for integration with telecom infrastructure (*46*); electro-optics will provide rapid tunability (*47*); adaptive optics will enable yet-higher *NA* operation (*48*); and nanophotonics in the end-mirror will enable direct integration with waveguide devices.

Last, in combination with microlens arrays to stabilize an array of waists, it should be possible to extend the small-waist resonator technique demonstrated in this paper to arrays of resonators spaced by a few microns, for immediate integration with Rydberg atom arrays (*49*, *50*). Crucially, because atoms can be millimeter-to-centimeter distances from the nearest optic, the sensitivity of Rydberg atoms to surface potentials is mitigated in these cavities compared to their nanophotonic counterparts. In short, this work heralds an era of optical cavity QED where strong light-matter coupling is widely available, rapidly extensible, and compatible with a much broader array of experimental platforms and technologies.

## MATERIALS AND METHODS

### Engineering an intracavity dipole trap by interfering the resonator’s longitudinal modes

We form our intracavity trap by driving the cavity with a far detuned 785-nm laser. Driving a single longitudinal mode of the cavity results in a one-dimensional optical lattice with a small waist, which after parity projection, typically leads to single atoms trapped at multiple sites of the lattice. Deterministic trapping of only one atom coupled to the cavity mode requires an optical tweezer potential—a dipole trap with a small waist. To create this dipole trap, we drive multiple longitudinal modes of the cavity by phase modulation of the trapping light. Since different longitudinal modes are phase shifted (in space) with respect to each other, choosing appropriate phase modulation frequency and strength can result in large suppression of the sinusoidal intensity variation across a finite region (*51*). For our cavity, we require this region to be centred at *d* = 1 cm from the curved mirror—the location of the small waist. Phase modulation with an electro-optic modulator at *n* times the FSR results in a trapping potential given by (to the first order)

$$U=U_{0}\left[J_{-1}{\left(\beta \right)}^{2}{\text{sin}}^{2}\left(kz-\frac{nd}{L}\pi \right)+J_{0}{\left(\beta \right)}^{2}{\text{sin}}^{2}\left(kz\right)+J_{1}{\left(\beta \right)}^{2}{\text{sin}}^{2}\left(kz+\frac{nd}{L}\pi \right)\right]$$ (1)

where *U*_0_ is the depth of the initial optical lattice, *k* is the wave vector of the carrier, β is the modulation depth, *L* ≈ 30 cm is the length of our cavity, and *J*_α_ is the Bessel function of first kind. We note the following identity

$${\text{sin}}^{2}\left(\mathrm{kz}-\frac{10}{30}\pi \right)+{\text{sin}}^{2}\left(\mathrm{kz}\right)+{\text{sin}}^{2}\left(\mathrm{kz}+\frac{10}{30}\pi \right)=\frac{3}{2}$$ (2)

For our cavity *L*/*d* ≈ 30, thus driving the *n* = 10 sidebands at equal intensity as the carrier [i.e., *J*_−1_(β)^2^ = *J*_0_(β)^2^ = *J*_1_(β)^2^], results in a cancellation of the sinusoidal potential at the cavity waist resulting in a dipole trap with depth *U*_0_/2 (see fig. S3). While this seems to imply that such a cancellation is only achievable with a fine tuning of the length and the waist location, numerical calculations suggest that it is possible for any location by changing the modulation depth. Note that the effect of higher-order sidebands can be similarly compensated for by slightly changing the modulation depth.

### Aligning readout/trapping lattice alignment to maximize light-matter coupling

Achieving the strongest possible coupling between the atom and the cavity requires that the atom be trapped at the 780-nm cavity mode antinode. We achieve this by using an intracavity lattice for trapping the atom and choosing the frequency of the lattice laser such that the antinodes of lattice standing wave and the cavity mode are approximately aligned at *z* = *d*, where *d* ≈ 1 cm is the distance of the mode antinode closest to the cavity waist, from the curved mirror. Note that we cannot guarantee that there is an antinode at the small waist location. At worst, the antinode is λ/4 away from the waist, which only changes the coupling by 0.5%.

To see how we find the correct lattice frequency, note that the lattice standing wave is given by the function ${\text{sin}}^{2}\left(\frac{\mathrm{nd}}{L}\right)$, where *n* is the order of the mode at which the lattice laser is resonant with the cavity, i.e., *L* = *n*λ/2 and λ ≈ 785 nm. Moving this standing wave one period requires changing the frequency by Δ*n*×FSR, where $\Delta n=\frac{L}{d}$. Our measured FSR gives *L* = 31.2 cm, so *L*/*d* is not an integer and therefore perfect alignment is not generally possible. But this guarantees that moving the frequencies by 16 FSRs would either pass through either a minimum or maximum of coupling.

One of the ways we keep track of this coupling is by measuring the VRS. Another, faster way to keep track of this coupling is to trap the particle in the dipole trap with a small residual standing wave, which still leads to parity projection but slightly increases the probability of finding an atom at the residual lattice intensity antinodes. This leads to change in scattering rate in the dipole trap as the lattice laser is moved through the different longitudinal modes. We have used both methods at different times.

We then use a binary search like procedure to find the minimum of the coupling, since that is a clearer signal than a maximum and then change the frequency by 16 FSRs to find the maximum. Last, we note that since smallest step that we can change the phase of the standing wave is $\frac{d}{L}\pi$, theoretically, the worst possible phase offset (if we perform our alignment procedure perfectly) we can have is $\frac{d}{2L}\pi =0.016\pi$, which corresponds to a misalignment of ≈6 nm.

### Molasses imaging and photon counting performance

At the cavity location, we use a pair of retro-reflected molasses beams for PGC. Each beam is detuned 40 MHz from the *F* = 2 → *F* = 3 transition with a slight power imbalance between the two paths (0.024 W/cm^2^ versus 0.015 W/cm^2^). We compensate for magnetic field gradients with three out-of-vacuum bias coils to operate at 0 field. The same parameters are used for PGC/light assisted collisions to load single atoms as for fluorescence imaging.

In terms of backgrounds, it is worth noting that without the atoms (but with the molasses light on) the dark count rate is 1.3 kHz. With the molasses light off, we see 750 Hz of background, corresponding to SPCM dark counts.

### Single atom lifetime

To characterize the lifetime of our trapped single atoms, we observe the atom while under molasses cooling light for 200 ms and plot average fluorescence level versus hold time. An exponential fit then yields a lifetime of 146 (6) ms, slightly longer than the short time survival measurement in Fig. 4. See fig. S4.

The atom lifetime without the molasses light (in the dark) fits to a lifetime of 50 ms. The nearly threefold increase in lifetime in the presence of molasses light is likely indicative of intensity noise–induced parametric heating in the cavity dipole trap/lattice.

### Experimental conditions for *g*_2_(τ) dataset

The *g*_2_(τ) dataset was collected at high outcoupling (*T* = 20% per round-trip) to maximize the data rate, using a pair of SPCMs to minimize the impact of detector afterpulsing (*52*) and time-tagged using custom firmware running on a RedPitaya built on top of the Zynq Time-to-Digital package [(*53*) and https://github.com/madamic/zynq_tdc].

### Error and uncertainty calculations

Error propagation for quoted parameters is carried out using Python’s uncertainties package, with error for fitted parameters provided by the covariance matrix. For uncertainty bars, two methods are used: (i) chi-squared, fit-based for the lifetime data and (ii) bootstrapping for the survival rate and fidelity data. For bootstrapping, we split the data into three sets, individually calculate relevant quantities, and then calculate the average and SD.

## Supplementary Materials

### This PDF file includes:

Supplementary Text Figs. S1 to S9 References
