# Supplementary material for: Cavity QED in a high NA resonator
Source: Sci Adv. 2025 Feb 26;11(9):eads8171. doi: 10.1126/sciadv.ads8171 (PMC11864187; doi:10.1126/sciadv.ads8171)
Supplement: Supplementary file 1 — Supplementary Text Figs. S1 to S9 References [file sciadv.ads8171_sm.pdf]

Science Advances
AAAS

Supplementary Materials for

Cavity QED in a high NA resonator

Danial Shadmany *et al.*

Corresponding author: Danial Shadmany, shadmany@stanford.edu

Sci. Adv. **11**, eads8171 (2025)
DOI: 10.1126/sciadv.ads8171

This PDF file includes:

Supplementary Text
Figs. S1 to S9
References

5 Supplementary Text

5.1 Derivation of Out-scattering Efficiency

For a cavity with cooperativity C_{max} in the absence of any outcoupling (just intracavity losses), the question we want to address here is how much outcoupling to *add* to maximize the fraction of photons scattered by the atom that leak out of the cavity: if we outcouple too little, more of the photons are lost to intracavity losses; if we outcouple too much we reduce the cooperativity and more photons are lost to free-space scattering.

Suppose we outcouple a factor β more photons than are lost intracavity. This provides a cavity outcoupling fraction $\frac{\beta}{1+\beta}$ and a cavity cooperativity $C = \frac{C_{max}}{1+\beta}$ The fraction of photons scattered by the atom that leak out of the cavity for detection is then $\chi = \frac{\beta}{1+\beta} \times \frac{C_{max}/(1+\beta)}{1+C_{max}/(1+\beta)}$. Maximizing this over β yields $\beta = \sqrt{1 + C_{max}}$, and $\chi = 1 - 2\frac{\sqrt{1+C_{max}}-1}{C_{max}}$.

In the limit of large maximum cooperativity $C_{max} \gg 1$, we then find that $C \approx \sqrt{C_{max}}$ and $\chi \approx 1 - \frac{2}{\sqrt{C_{max}}}$; on the other hand, in the limit of small cooperativity $C_{max} \ll 1$, we find that $C \approx \frac{C_{max}}{2}$ and $\chi \approx \frac{C_{max}}{4}$.

That is, in the limit of large C_{max} we should outcouple enough to reduce the cooperativity to the square root of its maximum value, resulting in outcoupling which is $\approx \sqrt{C_{max}}$ times larger than the intracavity losses. By contrast small C_{max} we should set our outcoupling equal to our intracavity losses, halving the cooperativity.

These results are interesting because common knowledge (see SI 5.2) was that coherent transfer of photons between atoms has an infidelity (for $C \gg 1$) of approximately $\frac{2\pi}{\sqrt{C}}$, whereas scattering light into a cavity (thought of as an incoherent process) has an infidelity of approximately $\frac{1}{C}$; rather than attributing this difference to coherent vs incoherent scattering, it is now clear that the difference arises from *neglecting cavity outcoupling efficiency* in the latter case. This is valuable to know, because otherwise it would have appeared more efficient to pitch and catch quantum information between atoms in separate cavities rather than coherently transferring between atoms in the same cavity - it is now apparent that these two processes have the same (unfavorable) scaling with the maximum cooperativity of the resonator.

5.2 Cavity-Mediated Coupling Between Atoms

This section follows the approach outlined in ref. (*54*) to understand the coherent excitation transfer between *any* pair of three level emitters in a cavity, be they atoms, superconducting circuits, something else entirely: Suppose we have two emitters (a & b) in the same cavity and want to coherently couple a long-lived excitation (between states g & f) from one to the other via a cavity-stimulated Raman transition through an atomic excited state e, with the cavity coupled to the $f \leftrightarrow e$ transition, and the classical laser drive coupled to the $g \leftrightarrow e$ transition. The Hamiltonian is:

$$H = g\left[c^\dagger(\sigma_a^{fe} + \sigma_b^{fe}) + c(\sigma_a^{ef} + \sigma_b^{ef})\right] + \Omega(\sigma_a^{eg} + \sigma_b^{eg} + \sigma_a^{ge} + \sigma_b^{ge}) + (\delta_c + i\frac{\kappa}{2})c^\dagger c + (\Delta + i\frac{\Gamma}{2})(\sigma_a^{ee} + \sigma_b^{ee}) \tag{S1}$$

Here the cavity is detuned from Raman resonance by an amount δ_c, the Raman transition is detuned from the excited state by an amount Δ, g is the single photon/single emitter light-matter coupling strength on the $f \leftrightarrow e$ transition, Ω is the Rabi frequency for the laser driving the $g \leftrightarrow e$ transition, Γ & κ are the excited atom and cavity decay rates, $c\dagger/c$ are the field creation and annihilation operators, and σ_l^{pq} is the transition operator from state q to state p in atom l.

There are, of course, many pulse protocols to convert $|fg;0\rangle \leftrightarrow |gf;0\rangle$, including cavity-mediated Raman (*55*) and π-pulse (*56*) based swaps. In the presence of dominant cavity and excited atom loss, they all have the same optimal performance up to factors of order unity. For simplicity we will skip counter-intuitive driving and all other adiabatic approaches (though they are covered in Simon's thesis), and consider only a 4-photon Rabi process.

In that case we can adiabatically eliminate the e-states, resulting in an effective 2-photon Hamiltonian (neglecting second order shifts that can be compensated with cavity & laser detunings):

$$H_2^{eff} = \frac{g\Omega}{\Delta}\left[c^\dagger(\sigma_a^{fg} + \sigma_b^{fg}) + c(\sigma_a^{gf} + \sigma_b^{gf})\right] + i\frac{\Omega^2}{\Delta^2}\Gamma(\sigma_a^{ff} + \sigma_b^{ff}) + (\delta_c + i\kappa/2 + i\frac{g^2}{\Delta^2}\Gamma)c^\dagger c \tag{S2}$$

Further eliminating the cavity mode results in an effective 4-photon Hamiltonian (neglecting fourth order shifts that can be tuned out):

$$H_4^{eff} = \frac{g^2\Omega^2}{\Delta^2\delta_c}\left[\sigma_a^{fg}\sigma_b^{gf} + \sigma_a^{gf}\sigma_b^{fg}\right] + i\left[\frac{\Omega^2}{\Delta^2}\Gamma + \frac{g^2\Omega^2}{\Delta^2\delta_c^2}(\kappa/2 + \frac{g^2}{\Delta^2}\Gamma)\right](\sigma_a^{ff} + \sigma_b^{ff}) \tag{S3}$$

A full flip-flop of an excitation between atoms a and b thus requires a time $\tau = \pi/\left(\frac{g^2\Omega^2}{\Delta^2\delta_c}\right)$, resulting in a total decoherence induced loss $\epsilon = \tau \times \left[\frac{\Omega^2}{\Delta^2}\Gamma + \frac{g^2\Omega^2}{\Delta^2\delta_c^2}(\kappa/2 + \frac{g^2}{\Delta^2}\Gamma)\right]$. In the limit $\Delta \to \infty$, this becomes $\epsilon = \pi\frac{2\Gamma\delta_c^2 + g^2\kappa}{2g^2\delta_c}$. This loss is minimized for $\delta_c = \frac{g\sqrt{\kappa}}{\sqrt{2\Gamma}} = \kappa\sqrt{\frac{C}{8}}$, with a value $\epsilon = \frac{2\pi}{\sqrt{2C}}$.

Because any two-atom gate can be implemented by (*55*) mapping two physically separate qubits into one four-level atom via the cavity field, performing internal four-level operations on the atom, and then mapping back to separate physical qubits via the cavity field, ϵ sets a lower limit on the infidelity of such a gate.

Similarly, the number of information exchanges between atoms, or between atom and cavity field, is $1/\epsilon \propto \sqrt{C}$.

Finally, a bit of interpretation: if we go to too large a detuning from the cavity, the 4-photon Rabi oscillation slows down too much and we are dominated by excited-state loss; if we go to too small a detuning from the cavity, the 4-photon Rabi oscillation speeds up, but not as much as the cavity leakage, and we are dominated by that. The sweet spot is in the middle. The excited state detuning, as long as it is large enough, does not contribute at all. Indeed one can show that even on resonance with excited state a counter-intuitive pulse sequence gives the same performance up to a numerical factor of order unity (see ref. (*54*)).

5.3 Cavity Optics Setup

See Figure S2 for a full schematic, including intra-cavity optics, photo-detectors, and in-coupling paths. The setup includes an EOM and a pellicle beam splitter inserted into the long propagation arm of the cavity. These are used for high-bandwidth cavity stabilization and tunable outcoupling respectively. During operation, we tune the angle of the pellicle to switch between 4% and 20% pick-off, depending on if we are optimizing for atom-light coupling (as for the measurement of the vacuum Rabi splitting) or overall photon collection efficiency (as for the readout histograms). The pellicle reflects light out on both sides, requiring the use of two SPCMs to maximize collection. Using the same pellicle, we also pick off 2% of the 785 nm trap light for a low-bandwidth, slow locking path, from which we generate an error signal and feedback to both an out of vacuum piezo on the end mirror and to the EOM.

In terms of robustness and technical overhead, operating at lower finesse has clear advantages. First, we can use relatively cheap optics without expensive HR coatings in our cavity (the C140 asphere used in the experiment was purchased from Thorlabs for $ 60). Second, gradual degradations of finesse and the deposition of hydrocarbons which can lead to increased cavity loss over time do

not affect the performance of our system. Finally, because we can operate the cavity with relatively high loss while achieving state of the art performance, we can have a vacuum window inside the cavity and operate with half of the cavity out of vacuum, allowing us to replace the planar end mirror as needed or insert additional optics to tailor the resonator as required (such as the EOM for high-bandwidth locking) without the time cost of breaking vacuum

5.4 Intracavity Dipole Trap from Multichromatic Driving

We form our intra-cavity trap by driving the cavity with a far detuned 785 nm laser. Driving a single longitudinal mode of the cavity results in a one-dimensional optical lattice with a small waist, which after parity projection, typically leads to single atoms trapped at multiple sites of the lattice. Deterministic trapping of only one atom coupled to the cavity mode requires an optical tweezer potential – a dipole trap with a small waist. To create this dipole trap, we drive multiple longitudinal modes of the cavity by phase modulation of the trapping light. Since different longitudinal modes are phase shifted (in space) with respect to each other, choosing appropriate phase modulation frequency and strength can result in large suppression of the sinusoidal intensity variation across a finite region (*51*). For our cavity, we require this region to be centred at $d = 1$ cm from the curved mirror – the location of the small waist. Phase modulation with an EOM at n times the free spectral range (FSR) results in a trapping potential given by (to the first order):

$$U = U_0 \left(J_{-1}(\beta)^2 \sin^2(kz - \frac{nd}{L}\pi) + J_0(\beta)^2 \sin^2(kz) + J_1(\beta)^2 \sin^2(kz + \frac{nd}{L}\pi) \right) \tag{S4}$$

where U_0 is the depth of the initial optical lattice, k is the wave-vector of the carrier, β is the modulation depth, $L \approx 30$ cm is the length of our cavity, and J_α is the Bessel function of first kind. We note the following identity :

$$\sin^2(kz - \frac{10}{30}\pi) + \sin^2(kz) + \sin^2(kz + \frac{10}{30}\pi) = \frac{3}{2} \tag{S5}$$

For our cavity $L/d \approx 30$, thus driving the $n = 10$ sidebands at equal intensity as the carrier (i.e. $J_{-1}(\beta)^2 = J_0(\beta)^2 = J_1(\beta)^2$), results in a cancellation of the sinusoidal potential at the cavity waist resulting in a dipole trap with depth $U_0/2$ (see Fig. S3). While this seems to imply that such a cancellation is only achievable with a fine tuning of the length and the waist location, numerical

calculations suggest that it is possible for any location by changing the modulation depth. Note that the effect of higher order sidebands can be similarly compensated for by slightly changing the modulation depth.

5.5 Aligning the 780 nm cavity mode and 785 nm intra-cavity lattice

Getting the strongest possible coupling between the atom and the cavity requires that the atom be trapped at the 780 nm cavity mode anti-node. We achieve this by using an intra-cavity lattice for trapping the atom, and choosing the frequency of the lattice laser such that the anti-nodes of lattice standing wave and the cavity mode are approximately aligned at $z = d$, where $d \approx 1$ cm is the distance of the mode anti-node closest to the cavity waist, from the curved mirror. Note that we can't guarantee that there is an anti-node at the small waist location. At worst, the anti-node is $\lambda/4$ away from the waist, which only changes the coupling by 0.5%.

To see how we find the correct lattice frequency, note that the lattice standing wave is given by the function $\sin^2(\frac{nd}{L})$, where n is the order of the mode at which the lattice laser is resonant with the cavity, i.e. $L = n\lambda/2$, $\lambda \approx 785$ nm. Moving this standing wave one period requires changing the frequency by $\Delta n \times$FSR, where $\Delta n = \frac{L}{d}$. Our measured FSR gives $L = 31.2$ cm, so L/d is not an integer and therefore perfect alignment is not generally possible. But this guarantees that moving the frequencies by 16 FSRs would either pass through either a minimum or maximum of coupling.

One of the ways we keep track of this coupling is by measuring the VRS. Another, faster way to keep track of this coupling, is to trap the particle in the dipole trap with a small residual standing wave, which still leads to parity projection, but slightly increases the probability of finding an atom at the residual lattice intensity anti-nodes. This leads to change in scattering rate in the dipole trap as the lattice laser is moved through the different longitudinal modes. We have used both methods at different times.

We then use a binary search like procedure to find the minimum of the coupling, since that is a clearer signal than a maximum and then change the frequency by 16 FSRs to find the maximum. Finally, we note that since smallest step that we can change the phase of the standing wave is $\frac{d}{L}\pi$, theoretically the worst possible phase offset (if we perform our alignment procedure perfectly) we can have is $\frac{d}{2L}\pi = 0.016\pi$, which corresponds to a misalignment of ≈ 6 nm.

5.6 Imaging Parameters and Histogram Analysis

At the cavity location, we use a pair of retro-reflected molasses beams for polarization gradient cooling. Each beam is detuned 40 MHz from the $F = 2 \rightarrow F = 3$ transition with a slight power imbalance between the two paths (0.024W/cm^2 vs .015 W/cm^2). We compensate for magnetic field gradients with three out-of-vacuum bias coils to operate at 0 field. The same parameters are used for PGC/light assisted collisions to load single atoms as for fluorescence imaging.

The width of the histogram recorded after imaging is set by the interplay of numerous effects. First is the shot noise of the light, where the variance naturally scales as a square root of the the signal amplitude. The finite temperature of the atom will also lead to a sampling of the different coupling rates at different points in the cavity, but for large photon numbers the distributions can be approximated as Gaussians and summed accordingly. Comparing between the lattice and dipole trap histograms, this already would predict a wider peak for the lattice, given that the signal is higher. Additionally, the procedure by which we transfer a single atom in the dipole trap to one in the lattice is not adiabatic and can impart a momentum kick to the atom such that it is trapped in an antinode away from the small waist and thus with lower signal, effectively broadening the spread in fluorescence further. In future iterations of the experiment, sideband cooling the atom in combination with adiabatically turning on the lattice might be implemented to narrow the width.

In terms of backgrounds, it is worth noting that without the atoms (but with the molasses light on) the dark count rate is 1.3 kHz. With the molasses light off we see 750 Hz of background, corresponding to single photon counter module (SPCM) dark counts.

5.7 Single Atom Lifetime

To characterize the lifetime of our trapped single atoms, we observe the atom while under molasses cooling light for 200 ms and plot average fluorescence level vs hold time. An exponential fit then yields a lifetime of 146(6) ms, slightly longer than the short time survival measurement in Fig 4. of the main text. See Figure S4.

The atom lifetime without the molasses light (in the dark) fits to a lifetime of 50 ms. The nearly threefold increase in lifetime in the presence of molasses light is likely indicative of intensity-noise-induced parametric heating in the cavity dipole trap/lattice.

5.8 Experimental Conditions for $g_2(\tau)$ dataset

The $g_2(\tau)$ dataset was collected at high outcoupling ($T = 20\%$ per round-trip) to maximize the data rate, using a pair of single photon counters (SPCMs) to minimize the impact of detector afterpulsing (*52*), and time-tagged using custom firmware running on a RedPitaya built on top of the Zynq Time-to-Digital package ((*53*) and `https://github.com/madamic/zynq_tdc`).

5.9 Cavity Stability and Transverse Mode Splitting

We calculate the waist size and Guoy phase of the small-waist resonator in the usual way, by taking the eigenvalues and vectors of the round trip ABCD matrix. Expressed as (1,q), the eigenvector provides the complex beam parameter q from which the waist size at a given cavity length can be extracted. The stability condition $\frac{f^2}{L_{long}-f} > L_{short} - f - R > 0$ is extracted by setting the norm of the eigenvalue equal to 1. The argument of this round trip eigenvalue provides the Guoy phase, or transverse mode splitting. We show plots of the resonator waist size and Guoy phase at the operating point of our cavity, where we set the long arm $L_{long} = 30$ cm and the focal length $f = 1.45$ mm for the C140TMD-B asphere. The only varying parameter then is the length of the short arm (distance between C140 and curved mirror). We plot the waist size against the perturbation in this short arm length from f (with f chosen as the edge of the stability diagram and the "design" distance of a normal lens system). See Figure S5.

It is worth noting that the small waist in Figure S5a occurs at the center of the stability diagram. This is the salient feature of our resonator geometry, indicating that a sub-micron mode waist can be achieved at the location of the atom without operating at the edge of the stability range, as is required in concentric cavities. It is also worth noting at this point that the Guoy phase for this resonator does not follow the usual bounds for a two mirror cavity. There, the Guoy phase and consequently the transverse mode splitting runs from 0 to 2π across the edges of the stability region. In the small waist geometry sketched here, the phase range is halved, running only from π on one end to 0. This is due to the asymmetry of the cavity. At the operation point of our cavity - the center of the stability diagram - the Guoy phase of the resonator is $\frac{\pi}{2}$. This has the favorable property that neither the first or second order modes are overlapped with the fundamental mode of the cavity, circumventing the mode-mixing issues of the confocal and concentric designs.

5.10 Concentric vs Lens Cavity Stability

Lens cavities are impressively more stable than concentric cavities. Let χ represent the sensitivity of cavity axis tilt to optic displacements. For fixed cavity waist w and focal length f, this ratio of χ between the two cavity types is given by

$$\frac{\chi_{concentric}}{\chi_{lens}} = \alpha \frac{x}{1 - \sqrt{1 - x^2}} \tag{S6}$$

where $\alpha > 1$ is a factor of order unity and $x = \frac{\pi w^2}{\lambda f}$ is a dimensionless quantity akin to the Fresnel λf number comparing the waist to the geometric mean of the wavelength and the focal length. For a 1 μ m waist at λ = 780 nm using a typical focal length of 10 mm, this ratio equals ≈5000. A two-lens cavity with these parameters is thus 5000× less sensitive to transverse optic displacements than a concentric cavity, dramatically alleviating the stringent stabilization requirements for operating a two-mirror cavity very close to concentricity. There are several other benefits that we will briefly mention here. Unlike concentric cavities, there is no inherent “last” stable resonance before leaving the stability diagram. Instead, by having multiple intracavity distances to tune, the optics can simply be placed for whatever arrangement produces the waist size desired. Additionally, convex optics are more substantially more manufacturable than concave optics at high curvature. This enables lower-aberration wavefronts entering the high-NA regime, which is crucial to actually achieving small design waists in the nonparaxial regime.

5.11 Error and Uncertainty Calculations

Error propagation for quoted parameters is carried out using Python’s uncertainties package, with error for fitted parameters provided by the covariance matrix. For uncertainty bars, two methods are used: (1) chi-squared, fit-based for the lifetime data, and (2) bootstrapping for the survival rate and fidelity data. For bootstrapping, we split the data into three sets, individually calculate relevant quantities, and then calculate the average and standard deviation.

5.12 $g^{(2)}(\tau)$ Calculation

There are three relevant timescales in this system : (i) the fastest one set by the Rabi frequency in PGC, detuning, atomic decay rates and the cavity lifetime, (ii) the time scale set by the scattering rate in the PGC, and (iii) the slowest timescale, set by the motion in the trap. While it might seem that the fastest, quantum mechanical timescale would be most interesting, it has been thoroughly explored in the context of resonance fluorescence. With time-resolved explorations of laser-cooling in mind, we are interested in the slowest of these timescales. Here the dynamics of the atom are entirely described by the classical Langevin equation of a harmonic oscillator. The 1D Langevin equation is of the form :

$$\frac{d^2x(t)}{dt^2} + \gamma\frac{dx(t)}{dt} + \omega^2 x(t) = \eta(t) \tag{S7}$$

Here $\eta(t)$ is the random force (noise term) that obeys $\eta(t)\eta(t') = \frac{2\gamma k_B T}{m}\delta(t-t')$, γ is the damping rate, m is the mass, ω is the oscillator angular frequency, and T is the temperature.

Our program for analyzing these dynamics is as follows:

1. Treat the atom as moving in a (separable) intra-cavity potential $U(x,y,z) = \frac{U_0}{1+\left(\frac{z}{z_r}\right)^2} \times \exp\left(-\frac{2(x^2+y^2)}{w_0^2(1+(z/z_r)^2)}\right) \approx U_0 \times \left(1-(\frac{z}{z_r})^2\right) \times \left(1-2\frac{x^2+y^2}{w_0^2}\right) \approx U_0 \times \left(1-(\frac{z}{z_r})^2 - 2\frac{x^2+y^2}{w_0^2}\right)$, where $z_r \equiv \frac{\pi w_0^2}{\lambda} \approx 3\ \mu$m is the Rayleigh range of the cavity mode, $w_0 \approx 930$ nm is the mode waist, and $\lambda \approx 780$ nm is the wavelength of the trapping light.

2. Treat the atomic scattering rate as separable in the Cartesian atom coordinates $I_{sc} \approx I_{pk} \times \cos^2 kz \times e^{-2(x^2+y^2)/w_0^2}$. Note that where the *trap* varies in z on a length scale of z_r, the scattering rate varies in z on a much shorter length-scale of $k^{-1} = \frac{\lambda}{2\pi}$ – by contrast, the radial trapping and radial scattering rates both vary on the length scale of w_0 - it is for this reason that we claim that the variations in fluorescence rate in the dipole trap arise predominantly from axial motion, at least insofar as axial and radial temperatures are equal.

3. Generate the equations of motion for each of the spatial degrees of freedom of the atom, including velocity dependent damping and Langevin noise term arising from the cooling beams (itself normalized based upon the axis-dependent temperature).

4. For the radial direction, solve these equations for the two-time correlators of each degree of freedom of the atom. For the axial direction, the fast $\cos^2(kz)$ variation of the scattering rate requires calculation of the the marginal probability distribution $P(z,t)$

5. Employ these two-time correlators and the marginal distribution of the atomic position, in conjunction with the knowledge that since the equations of motion are linear the motional degrees of freedom act as gaussian processes, to compute two-time correlators of the intensity. We will find that the two-time intensity correlator factorizes into independent correlators in x,y, and z.

6. Normalize the two-time correlator using the expected value of the intensity.

We first note that since motion in the three directions is approximately independent, the variations of scattering rates due to motion in the three directions are uncorrelated. Writing scattered intensity as $I(t) = I_{pk}I_x(x(t))I_y(y(t))I_z(z(t))$, we find :

$$\begin{aligned} g^{(2)}(\tau) &= \frac{I_x(x(\tau))I_y(y(\tau))I_z(z(\tau))I_x(x(0))I_y(y(0))I_z(z(0)}{I_x(x(0))I_y(y(0))I_z(z(0)^2} \\ &= \frac{I_x(x(\tau))I_x(x(0))}{I_x(x(0))^2}\frac{I_y(y(\tau))I_y(y(0))}{I_y(y(0))^2}\frac{I_z(z(\tau))I_z(z(0))}{I_z(z(0))^2} \\ &= g_x^{(2)}(\tau)g_y^{(2)}(\tau)g_z^{(2)}(\tau) \end{aligned} \tag{S8}$$

5.13 The radial part

In one of the radial directions, say x, the scattered intensity varies as $I_x(x) = e^{-\frac{2x^2}{w_0^2}} \approx 1 - \frac{2x^2}{w_0^2}$. This implies :

$$g_x^{(2)}(\tau) = \frac{\left\langle \left(1 - \frac{2x(\tau)^2}{w_0^2}\right)\left(1 - \frac{2x(0)^2}{w_0^2}\right)\right\rangle}{\left\langle 1 - \frac{2x(0)^2}{w_0^2}\right\rangle^2} \tag{S9}$$

We assume a gaussian process and use Wick's theorem to simplify the fourth order correlator and obtain:

$$g_x^{(2)}(\tau) = 1 + \frac{8}{w_0^4} \frac{x(\tau)x(0)^2}{\left(1 - \frac{2x(0)^2}{w_0^2}\right)^2} \tag{S10}$$

We further note that for a particle governed by equation S7, the second order position correlator is well known and given by Ref (*57*):

$$x(\tau)x(0) = \frac{k_B T_r}{m\omega_r^2} e^{-\frac{\gamma_r}{2}|\tau|} \left(\cos(\omega'\tau) + \frac{\gamma_r}{2\omega'} \sin(\omega'|\tau|)\right), \tag{S11}$$

where T_r is the radial temperature, ω_r is the radial trap angular frequency, γ_r is the radial damping rate, and $\omega' = \sqrt{\omega_r^2 - \gamma_r^2/4}$. This completes the calculation for $g_x^{(2)}(\tau)$. We assume that our system has cylindrical symmetry, so $g_y^{(2)}(\tau) = g_x^{(2)}(\tau)$.

5.14 The axial part

In the axial direction z, the variation in scattered intensity is due to the standing wave nature of the mode, i.e. $I_z(x) = \cos^2(kz)$, leading to:

$$g_z^{(2)}(\tau) = \frac{\cos^2(kz(\tau))\cos^2(kz(0))}{\cos^2(kz(0))^2} \tag{S12}$$

5.14.1 Intra-cavity lattice

For an atom trapped in the intra-cavity lattice, we can follow the same procedure as the radial calculation i.e. Taylor expand the cosine, and apply Wick's theorem, yielding :

$$g_x^{(2)}(\tau) = 1 + 2k^4 \frac{z(\tau)z(0)^2}{\left(1 - k^2 z(0)^2\right)^2} \tag{S13}$$

5.14.2 Dipole trap

This is not possible for an atom in the dipole trap, which has a length scale given by z_r, the Rayleigh range. An atom could possibly sample even multiple longitudinal mode anti-nodes while staying in the trap.

Here we instead use the fact that the trap frequency in dipole trap is much lower, and assume that the motion of the particle is overdamped. This allows us to simplify the Fokker-Planck equation

and solve for the marginal probability distribution, $P(z,t;z_0)$, the probability of finding a particle at position z at time t given that the particle was at position z_0 at time $t = 0$. The Fokker-Planck equation simplifies to (*57*):

$$\frac{\partial P(z,t)}{\partial t} = \frac{\partial}{\partial z}\left(\frac{\omega_{ax}^2}{\gamma_{ax}} z P(z,t)\right) + \frac{k_B T_{ax}}{m\gamma_{ax}} \frac{\partial^2 P(z,t)}{\partial z^2} \tag{S14}$$

Here T_{ax} is the axial temperature, ω_{ax} is the axial trap angular frequency, γ_{ax} is the radial damping rate. Such a simplification and marginalization of the full distribution including velocity is possible because the particle attains terminal velocity at every position, giving $v = \frac{\omega_{ax}^2}{\gamma_{ax}} z$. This allows the inertial term in the Langevin equation to be neglected. For the initial condition $P(z,0) = \delta(z - z_0)$, the solution to this equation is a gaussian given by:

$$P(z,t;z_0) = \frac{1}{\sigma(t)\sqrt{2\pi}} \exp\left(-\frac{(z - z_0\alpha(t))^2}{2\sigma(t)^2}\right), \tag{S15}$$

with $\alpha(t) = e^{-\frac{\omega_{ax}^2}{\gamma_{ax}}t}$, $\sigma(t) = \sigma_0(1 - \alpha(t)^2)$ and $\sigma_0 = \frac{k_B T_{ax}}{m\omega_{ax}^2}$. $P(z,t;z_0)$ is thus the conditional probability of finding the atom at position z at time t, given that it was at position z_0 at time $t = 0$, exactly the distribution required to calculate two-time correlators. Furthermore, $P_\infty(z) = P(z,\infty;z_0)$ gives the probability of finding an atom at position z in steady state.

For the denominator of $g_z^{(2)}(\tau)$ we have:

$$\begin{aligned} \cos^2(kz)^2 &= \left(\int_{-\infty}^{\infty} dz P_\infty(z)\cos^2(kz)\right)^2 \\ &= \frac{1}{2}\left(1 + e^{-2k^2\sigma_0^2}\right)^2 \end{aligned} \tag{S16}$$

For the numerator we get:

$$\begin{aligned} \langle\cos^2(kz(\tau)\cos^2(kz_0)\rangle &= \int_{-\infty}^{\infty}\left(dz_0 P_\infty(z_0)\cos^2(kz_0)\int_{-\infty}^{\infty} dz P(z,t;z_0)\cos^2(kz(\tau))\right) \\ &= \frac{1}{4}\left(e^{-4k^2\sigma_0^2}\cosh\left(4\alpha(\tau)k^2\sigma_0^2\right) + 2e^{-2k^2\sigma_0^2} + 1\right) \end{aligned} \tag{S17}$$

Putting it all together and simplifying with a bit of algebra, we get for the dipole trap:

$$g_z^{(2)}(\tau) = 1 + \frac{2\sinh^2\ 2k^2\sigma_0^2 e^{-\left(\frac{\omega_{ax}^2}{\gamma_{ax}}|\tau|\right)}\Big)}{\left(e^{2k^2\sigma_0^2} + 1\right)^2} \tag{S18}$$

It is important to note at this point that the timescale for the g2 oscillations derived here correspond to correlations of the axial/radial motion of the atom in the trap, not the anti-bunching of photons emitted from a single atom, which occurs on a ns resolution. The plots in our paper are thus displayed on a different time scale than in earlier literature. In our system, it would be challenging to achieving the time resolution required to observe the anti-bunching of photons emitted from the single atom. This is because our primary measurements of the single atom are done in fluorescence 40 MHz detuned from atomic resonance. This adds fast oscillations to the usual g2 function with a period of $1/\Delta = \frac{1}{2\pi(40+20)\mathrm{MHz}} = 2.5$ ns. Achieving this timing resolution with the current performance of our FPGAs would require unrealistically long experiments.

5.15 Effect of temperature on average atom-cavity coupling

The geometric expression for the cooperativity mentioned in the main text is valid for a standing wave cavity with the atom perfectly localized to a cavity mode anti-node at the cavity waist. In reality, due to its finite temperature, the atom samples a range of different positions and therefore different couplings to the mode. This leads to an average observed cooperativity lower than the peak value. We note that we are neglecting the effect of the atom sampling different velocities, Doppler broadening the atomic transition. We expect this effect to be much smaller than the lowering of effective coupling, even at the highest expected temperatures.

The functional dependence of cooperativity on the position is exactly the same as that of scattered intensity discussed in SI 5.12. In fact, if the average value of the cooperativity is reduced from the peak cooperativity (C_{peak} by a factor f), i.e. $C = fC_{peak}$, the factor f is given by exactly the kind of integrals we have already calculated for the denominator of $g^{(2)}$ in SI 5.12. We are only interested in the cooperativity when the atom is trapped in the lattice. In this case the reduction factor is given by:

$$f = \left(1 - \frac{2x^2}{w_0^2}\right)\left(1 - \frac{2y^2}{w_0^2}\right)\left(1 - k^2 z^2\right) \tag{S19}$$

As before, x and y are the radial directions and z is the axial direction,

$$x^2 = y^2 = \frac{k_B T_r}{m\omega_r^2}$$
$$z^2 = \frac{k_B T_{ax}}{m\omega_{ax}^2} \tag{S20}$$

This gives,

$$f = \left(1 - \frac{k_B T_r}{2U_0}\right)^2 \left(1 - \frac{k_B T_{ax}}{2U_0}\right) \tag{S21}$$

Here U_0 is the depth of the trap. In this expression we have neglected the slight difference between 785 nm trap profile and the 780 nm mode profile and also assumed perfect alignment between both of them. For our measured values of $U_0/k_B \approx 800\ \mu$K and $T_r \approx T_{ax} \approx 200\ \mu$K, we get $f \approx 0.67$. Based on our waist, $w_0 = 930$ nm and finesse, $F = 40$, $C_{peak} = 5.6$. Accounting for another factor of 2 from lack of optical pumping and probing with a linear polarization, we predict $C = 1.87$, close to the measured value of 1.6. This difference could potentially be explained by the effect of stochastic loading of the atom in different sites along the z direction on the scale of a Rayleigh range, which would lead to additional variation in coupling, which we have neglected.

5.16 Mode Waist Measurement

Approaching the diffraction limit $1/kw_0 \rightarrow 1$, the smallest achievable cavity waist is influenced by several non-quadratic optical effects: non-paraxial propagation & aberrations of the asphere (*37*) and vector polarization effects (*58*) that are small in free space are enhanced by numerous cavity round-trips. Rather than relying exclusively on modeling to estimate the beam-waist, we prefer to measure it directly.

A traditional knife edge measurement of the waist is not possible, because once the losses from the edge become comparable to the external and internal losses of the cavity (long before it actually approaches the waist), the finesse drops precipitously. In addition, the short ($z_r \sim 3\ \mu$m) Rayleigh range of the sub-micron waist necessitates measurement with an object that is thin compared to z_r, impractical for a razor blade. Indeed, even SNOM-based mode-mapping techniques (*59*) result in too much loss near the waist of a sub-micron waist resonator.

We therefore introduce a way of measuring the mode waist of a cavity in situ using an end mirror with a small, sub-wavelength hole providing local outcoupling of the cavity field. The size of the hole is chosen such that the additional loss introduced is small compared to the cavity round trip

loss and the finesse does not drop appreciably for all measurement locations, while still providing enough outcoupling to detect the transmission. We use a gold coated SiN membrane (Norcada) with two holes of nanopores (of radius 100, 200, or 350 nm) separated by 7 μm. The gold coated membrane acts as the flat end mirror of the cavity which guarantees the focus to be right on the membrane because of the flat wavefront set by the boundary conditions. We carried out our test by using aspheric lens with $f = 1.5$ mm (Thorlabs C140 TMD-B), identical to what we used in the main text.

It is worth noting that the ABCD calculations for the cavity with (1) the planar mirror and (2) the curved mirror retro-reflector show the same waist size in the focal plane of the asphere. Intuitively, this is because the curved mirror acts as a 2f imaging system which re-images the waist to itself. The insertion of the curved mirror does not lead to non-paraxial aberrations, as a spherical mirror is an ideal reflector even for the non-paraxial case. For the measurement of the cavity waist through the membrane, the width of the fitted signal is a convolution of the actual cavity mode waist and the membrane hole, and therefore sets an upper bound on the waist of the small waist resonator.

The transmission through a sub-wavelength nanopore is approximated by the Bethe formula (*60*) $T \propto (\lambda/r)^4$ for a circular hole with radius r at wavelength λ in a thin and transversely infinite conductor. In practice the finite thickness of the gold deposition as well as the existence of surface plasmons and plasmon resonances around the hole complicate the calculation of the hole transmission; because of the r^{-4} dependence of transmission on hole size, the size must be carefully chosen to ensure that the transmission is smaller than the mirror transmission $T = 1 - 98.5\% = 0.015$ to avoid spoiling the cavity finesse, but large enough to yield a detectable signal. Experimentally we found a diameter of 200 nm gave a clear transmission signal while only reducing the cavity finesse by 15% from 52 to 42, whereas 350 nm introduced excess loss destroying the cavity and 100 nm yielded no transmission detectable above the noise floor. We expect that the impact of the 200 nm pinhole's size on our waist measurement to be ignorable. The hole is then scanned by a homebuilt, mechanically multiplied 2D piezo stage transverse to the cavity axis to determine the profile of the cavity waist. The well-defined separation of the two holes is used as an absolute calibration of the scanning setup.

In figure S7 the transmission through the nanopore is shown as a function of transverse position. The waist is determined by a gaussian fit to both holes.

We extract the waist size for several different positions in the stability diagram, as a function of mirror lens separation. We measure the finesse and amplitude by sweeping the laser over multiple FSRs at every transverse position. From the higher order modes in the transmission spectrum we infer the transverse mode spacing and the position in the stability diagram.

5.17 Comparing Lens and Cavity Light Collection Rates

We would like to compare the collection efficiency of a high numerical aperture lens (parameterized here by NA_{lens}) to that of a standing wave cavity with waist size also limited by NA_{lens}.

The first thing to note is that if all we care about is maximizing the cavity cooperativity and our loss comes entirely from clipping, it is *always* better to reduce the cavity NA by increasing the mode waist to improve the finesse. The idea is that the finesse limit is set by clipping four per round-trip to $F_{cav} = \frac{2\pi}{4e^{-2\mathrm{NA}_{lens}^2/\mathrm{NA}_{cav}^2}}$, where $\mathrm{NA}_{cav} = \frac{\lambda}{\pi w_0}$ and w_0 is the cavity mode waist. The cavity cooperativity is then: $C = \frac{24F}{\pi}\frac{1}{(kw)^2} = \frac{6F}{\pi}\mathrm{NA}_{cav}^2 = 3\mathrm{NA}_{cav}^2 e^{2\mathrm{NA}_{lens}^2/\mathrm{NA}_{cav}^2}$

Because the gaussian factor grows faster than the quadratic prefactor shrinks, the expressions counterintuitively tells us that we should go to low NA_{cav} (large cavity waist) to maximize the cooperativity.

However, this expression does not capture all considerations. For example, one might *want* a small mode waist for other reasons, like coupling to atoms in an array, or because achieving high cooperativity at small mode waist relaxes finesse requirements and speeds up readout. The upshot is that, if we do not expect to be able to get our finesse higher than a few thousand (for example due to material or linewidth constraints), choosing $\mathrm{NA}_{cav} \approx \frac{1}{2}\mathrm{NA}_{lens}$ is plenty, as it leads to a finesse of approximately 4600; increasing the cavity NA rapidly reduces the finesse – choosing $\mathrm{NA}_{cav} \approx \frac{3}{4}\mathrm{NA}_{lens}$ reduces the finesse to 54.

With the choice $\mathrm{NA}_{cav} \approx \frac{1}{2}\mathrm{NA}_{lens}$ out of the way, we now assume we actually end up with a substantially *lower* finesse limited by material constraints (glass absorption, imperfect AR coatings, etc...); in the absence of cavity outcoupling, the maximum achievable finesse is assumed to be F_{max}; choosing to an outcoupler with transmission β times the internal cavity losses reduces the finesse to $\frac{F_{max}}{1+\beta}$, but outcouples a fraction $\frac{\beta}{1+\beta}$ of the light within the cavity.

As such, if, in the absence of the cavity, an atom scatters into 4π at a rate $\Gamma_{4\pi}$, the cavity

will collect light and outcouple it at a rate $\Gamma_{cav} = \Gamma_{4\pi} \times \frac{6F_{max}}{\pi(1+\beta)} \times \mathrm{NA}_{cav}^2 \times \frac{\beta}{1+\beta}$. If we optimize this rate over β, we find that we should choose $\beta = 1$, halving the cavity finesse, resulting in $\Gamma_{cav} = \Gamma_{4\pi} \times \frac{3F_{max}}{2\pi} \times \mathrm{NA}_{cav}^2$. Noting that $\mathrm{NA}_{cav} \approx \frac{1}{2}\mathrm{NA}_{lens}$ we have: $\Gamma_{cav} = \Gamma_{4\pi} \times \frac{3F_{max}}{8\pi} \times \mathrm{NA}_{lens}^2$.

We should now compare this to the single-lens collection efficiency. A lens with $\mathrm{NA}_{lens} \ll 1$ subtends a solid angle $\Omega_{lens} \approx \pi\mathrm{NA}_{lens}^2$. This means that the lens collects light at a rate $\Gamma_{lens} = \Gamma_{4\pi} \times \frac{\Omega_{lens}}{4\pi} = \Gamma_{4\pi} \times \frac{\mathrm{NA}_{lens}^2}{4}$.

We can now compute the cavity collection efficiency enhancement $R = \frac{\Gamma_{cav}}{\Gamma_{lens}} = \frac{3F_{max}}{2\pi} \approx \frac{F_{max}}{2}$. That is, the total improvement in collection that the cavity provides is given by half of its finesse.

Note: We have neglected polarizations, assumed that cavity scattering does not induce extra heating, and worked for the lens in the low NA limit. These assumptions can be made most accurate with an optically pumped atom at a cavity anti-node, detected in cavity transmission.

5.18 Cavity Structure Mechanical Design

As covered in Supplement 5.9, the difficulty in operating cavities at the small waist points of the stability diagram arises from the increased sensitivity of the cavity axis to transverse misalignments. This sensitivity is much improved for the small waist cavity geometry outlined in this paper. Nonetheless, precise in-vacuum positioning is still necessary to account for drifts in cavity alignment during the bake and to tune the relative position of optics to the stable point. Our custom designed/machined flexure mounts allow us to accomplish this task without the use of commercial integrated three-axis piezo stages, which can have long lead times, less throw, and high cost.

The cavity mount uses two waterjet-cut stainless steel flexures for 3D control of the relative transverse alignment between the C140 asphere and the ROC = 1 cm curved mirror:

1. A single-axis vertical flexure mount for the curved mirror which provides control over the y-axis control
2. A two-axis horizontal flexure that attaches to the asphere mount and provides x/z motion

Both flexures employ a similar lever arm design to amplify the base 45 μm throw of the NAC2003-H32 Noliac piezo stacks. Initial designs used a straight lever arm, for which the theoretical amplification is given simply by the constrained ratio of displacements in piezo, set by the angle

of the arm. Space constraints motivated switching to the curved metal flexing elements shown in Figure S8. To simulate the predicted amplification for this design, we used Fusion's stress analysis software with factors included to account for the stress/strain parameters (Young's modulus, etc.) of stainless steel. We define our coordinate system as follows: x (perpendicular to both the cavity axis and transport directions), y (along transport directions), z (along the cavity axis). For the vertical mount, two piezos were used (one for each side to preserve symmetry), and we use a Mach-Zehnder interferometer to measure an amplified throw of $\sim 96\ \mu$m. The horizontal flexure operates in 2D, with mobile central platform supported by four lever arm slots. Only two of these four slots are mounted with piezo stacks – one for control in x and the other for z. We measure and amplified throw of 80 μm in both directions.

The completed stainless steel structure is secured using vented silver screws, with the vertical flexure sitting upright on the horizontal flexure. The entire assembly sits on top of a base plate to properly align the component relative to the loadlock translator arm. We use silver prism mirrors for the 3D molasses beams. These slide into custom machined molasses mounts (see Figure S8) with angles set to achieve cooling at the small waist.

With the flexure mount described above in vacuum, we achieve a pressure of 4×10^{-10} torr. Most if not all of the wires are insulated with kapton.

5.19 Model-Free Estimation of the Atom Detection Efficiency

The figures of merit for assessing the performance of the cavity-enhanced readout demonstrated in this paper are (1) the atom detection fidelity for a measurement of time t and (2) the survival rate of the atom during that measurement. To estimate these quantities, it is customary to tailor individual fitting routines to the separate sections of the single-atom histogram (background peak, atom peak, signal tails, etc). However, for fidelities above 99%, the extrapolated fidelity becomes highly dependent on the functional form of the histogram in its low probability wings (*61*). In a cavity the dependence of g on the axial position of the atom further complicate this choice of functional forms by introducing averaging of the dipole signal over the nodes and anti-nodes of the readout mode, or by creating an axial variation in coupling strength beyond the Raleigh range, relevant for lattice signals where the signal atom can in principle be localized one of many sites.

To circumvent this issue we employ a model-free estimation scheme for calculating the atom detection efficiencies, as detailed in Section 2.6.7 of reference (*61*). The method relies on extrapolating the survival rate S and fidelity F from the set of probabilities $p_{x_1 x_2}$, where x_1 is 0 if the the signal is measured above the threshold in the first of two consecutive shots, and x_2 defined similarly for the the second shot in the measurement. The probabilities $p_{x_1 x_2}$ can than be enumerated individually and related to F and S in a system of equations:

$$p_{11} = fF_1^2 S + (1-f)(1-F_0)^2 + fF_1(1-S)(1-F_0) \tag{S22}$$

$$p_{10} = fF_1 S(1-F_1) + fF_1(1-S)F_0 + (1-f)(1-F_0)F_0 \tag{S23}$$

$$p_{01} = f(1-F_1)SF_1 + f(1-F_1)(1-S)(1-F_0) + (1-f)F_0(1-F_0) \tag{S24}$$

where f is the filling fraction, and the overall fidelity $F = fF_1 + (1-f)F_0$. From the two shot data, it is easy to extrapolate f, p_{11}, p_{10}, and p_{01} at a given threshold and then to extrapolate F and S from the equation above. Generally, there will be two solutions, one of which can be discarded since as it will predict F ¡ 0.5. For fidelities up to 99.9%, the final F is robust to fluctuations in the loading fraction f, though the component fidelities F_0 and F_1 can be highly sensitive to it. There is only a narrow range of the loading fraction for which all solved parameters are physical, providing a check on the reasonableness of the extrapolated f.

5.20 Comparing Cooperativity Expressions

Here we begin with the most commonly used expression for cooperativity, $C \equiv \frac{4g^2}{\kappa\Gamma}$, and show that, up to numerical factors, it is equivalent to (a) the closed-transition cooperativity $C_{closed} = \frac{24F}{\pi}\frac{1}{(kw_0)^2}$, and (b) the Purcell factor, $F_P = \frac{3}{4\pi^2}\lambda^3\frac{Q}{V}$.

To show that $C = C_{closed}$, we note that for a closed transition, the atomic linewidth can be written using the Weisskopf-Wigner formula: $\Gamma \equiv \frac{\omega^3 d^2}{3\pi\epsilon_0\hbar c^3}$, where d is the dipole moment of the transition. The cavity linewidth can be written in terms of the cavity length L and finesse F according to $\kappa = 2\pi \times \frac{c}{2L} \times 1F$. Finally, the vacuum Rabi coupling g can be written as $g = dE_1/\hbar$, where E_1 is the electric field of a single photon in the cavity at the location of the atom. By

conservation of energy $\frac{1}{2}\epsilon_0 E_1^2 \times \frac{\pi}{2} w_0^2 L = \frac{1}{2}\hbar\omega$, so $g^2 = \frac{2d^2\omega}{\hbar\epsilon_0\pi w_0^2 L}$. Combining all of these results yields: $C \equiv \frac{4g^2}{\kappa\Gamma} = \frac{24F}{\pi} \times \frac{1}{(kw_0)^2} = C_{closed}$, as anticipated.

To show the equivalence of the Purcell factor F_P, we just note that for a standing wave resonator of length L and finesse F, the quality factor is given by $Q = \frac{F(2L)}{\lambda}$, and the mode volume by $V = \frac{1}{2}\frac{\pi}{2}w_0^2 L$. Combining these expressions yields $F_p = \frac{12F}{2\pi^3}\frac{\lambda^2}{w_0^2} = \frac{24F}{\pi}\frac{1}{(kw_0)^2}$, equal to C,C_{closed}.

In total, we can understand this equivalence to mean that, at least for atoms with closed transitions, the probability that the atom emits into a cavity is entirely independent of both transition's dipole moment, and length of the cavity. Atoms with narrow transitions (small dipole moments) emit slowly into cavities, but they emit into freespace *even more slowly*. Similarly, a long cavity has a small g, and hence a small light-matter coupling strength, but it also has a narrow linewidth, so the weak coupling has more time to coherently reinforce itself.

The only caveat to this story is that weak transitions and long cavities result in slower atom emission into the resonator, and hence *slower* readout; if readout speed is truly the currency, then choosing shorter cavities and stronger transitions is sensible.

5.21 Photon Loss Budget

The speed with which high-fidelity single atom detection can be performed is set by the photon collection rate at the photodetector, accounting for all loss channels in the system. We enumerate these for our system at the high outcoupling (20%) point:

1. **Cavity Collection:** 15%. At optimal Pellicle outcoupling, the cavity collects 15% of photons (see SI 5.1). This is double the free space collection efficiency 7.8 % of the ideal 0.56 NA of the C140. Our expression accounts for the birefringence of the cavity due to the EOM and Pellicle beamsplitter and for the delocalization due to the finite temperature of the atom. Without the birefringence the predicted collection efficiency increases to 25 %, and for an atom perfectly localized to an anti-node this further rises to 35 %.

2. **Fiber Coupling:** 50 %. Light that leaks out of the cavity on both sides of the Pellicle beamsplitter propagates past an 808 nm line filter, where locking and signal paths are divided, and through to the fiber incoupler. The 780 nm reflection of the LL01-808 Max Line filter

from AVR optics can be optimized to be effectively loss (R ¿ 99.9 %). We measure the fiber coupling efficiency of both paths to be $\approx$ 50%.

3. **Quantum Efficiency:** 64 %. We use single photon counter module SPCM-AQR-14, with a quantum efficiency of 64 % at 780 nm.

Overall, we calculate a total collection efficiency of 4.8 %. These parameters are far from optimized. With a more sophisticated locking and outcoupling scheme to obviate the need for birefringent intracavity optics together with the use of an EMCCD which does not require fiber-coupling and has a higher overall quantum efficiency (80 % at 780 nm), a fourfold increase in total collection efficiency to 20 % should be achievable. Assuming that detection time goes linearly with improvements in efficiency, this will enable readout with 99.5 % fidelity in 27 μs. Beyond these improvements, the use of lenses with better anti-reflection coatings should enable further gains in the photon collection rate of the cavity by increasing the finesse and therefore the Purcell factor. At present, we extrapolate an internal loss of the cavity of $\approx$ 12%, based on our measured finesse at 4% outcoupling. This loss includes the relatively poor anti-reflection coating on the C140-TMD-B asphere with round trip loss of 1.2 % in addition to loss due to clipping. Both issues can be circumvented with better polished aspheres. This will enable high-fidelity detection in 10 μs.

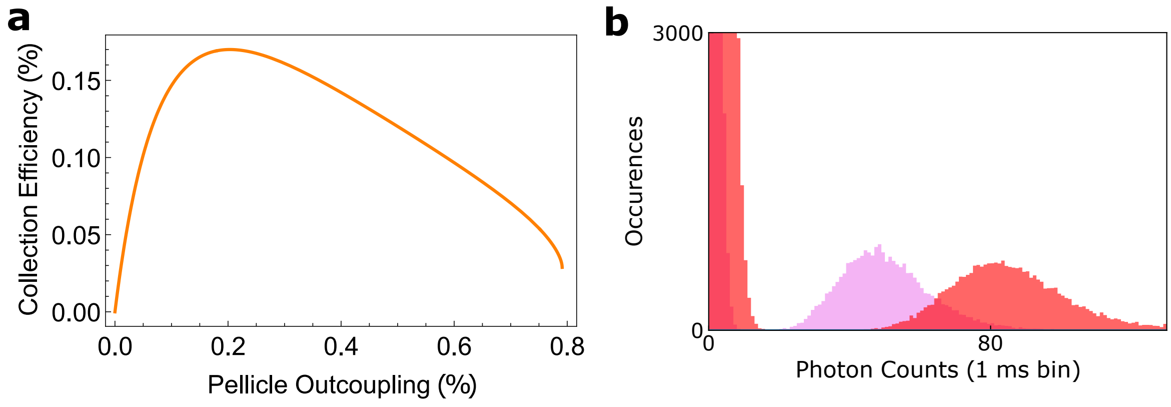

Figure S1: **Cavity Collection Efficiency. (a)** Photon collection efficiency as function of outcoupling fraction for measured cooperativity **(b)** histogram showing improvement between increase in outcoupling from 4% (pink) to 20% (red)

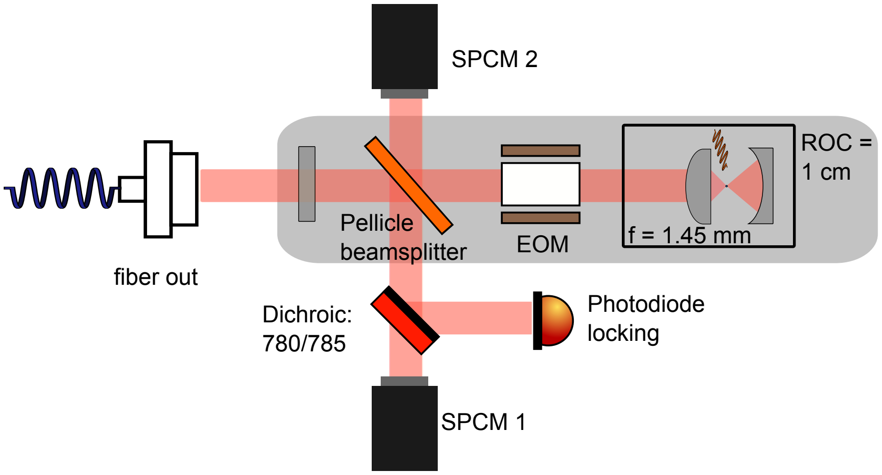

Figure S2: **Detailed Cavity Setup.** The resonator with all in-cavity optics is highlighted in gray. In addition to the aspheric lens, spherical mirror, and planar mirror, we also depict the pellicle used for outcoupling, and the intra-cavity electro-optic modulator that we use to lock the resonator with $\sim$ 100 kHz of bandwidth. Light outcoupled from both sides of the pellicle is coupled to single photon counting modules (SPCMs); a dichroic on one side of the outcoupler picks off the 785 nm locking light.

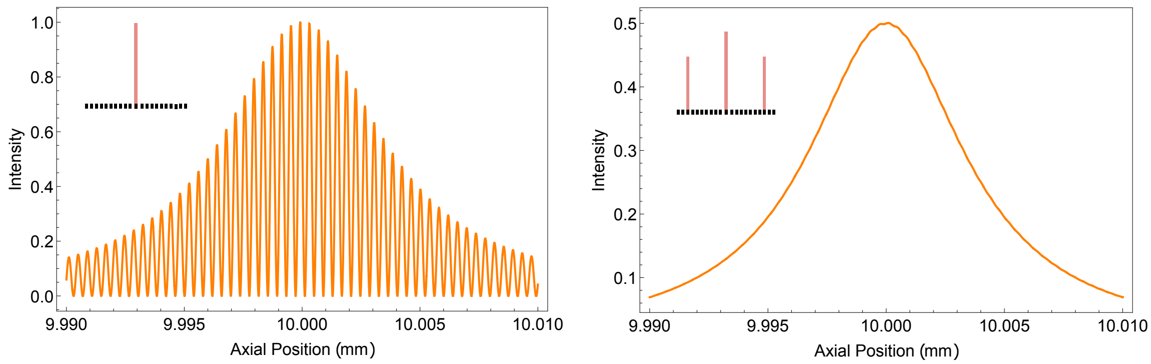

Figure S3: **Engineering the intra-cavity dipole trap. left** the intensity profile of a single longitudinal mode of the cavity, which takes the form of standing wave alternating with nodes and anti-nodes **(inset)** the lattice profile is generated by a single tone **right** a smooth intensity profile is required to trap a single atom using light assisted collisions at the small waist. To realize this in the cavity, we drive sidebands 10 FSRs from the carrier, tuning the relative power in the peaks until the multiple longitudinal modes interfere to create a dipole trap. **(inset)** the dipole trap is generated by the interference of a carrier with its sidebands

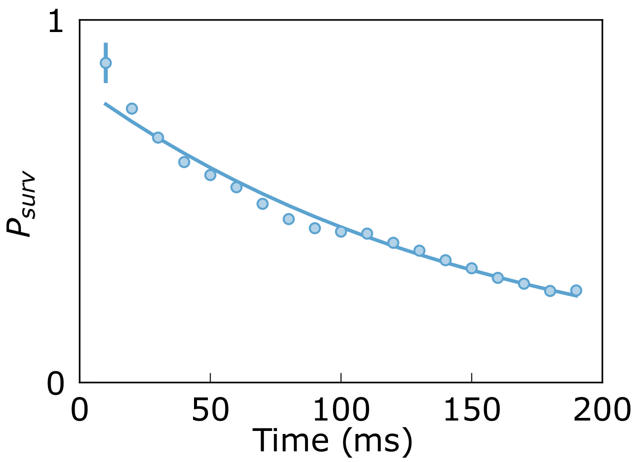

Figure S4: **The lifetime of the atoms.** Survival probability of single atoms in the trap vs hold time, characterized by average fluorescence level over 700 shots, with lifetime fitted to 146(6) ms.

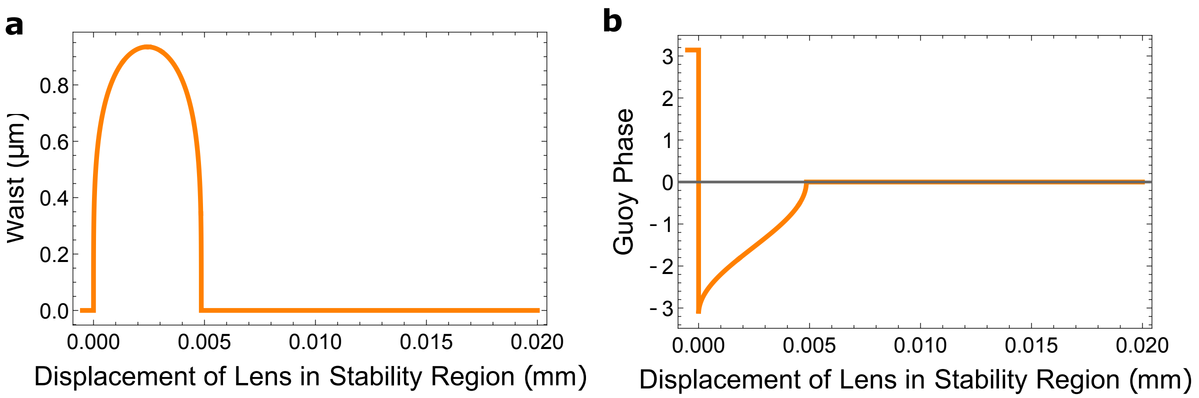

Figure S5: **The stability properties and diagram of the small waist resonator. a** the small waist cavity stability diagram with a long arm $L_{long} = 30$ cm. A waist of 930 nm is achieved at the center of the stability diagram **b** the Guoy phase of the cavity across the stability range, sweeping from π to 0.

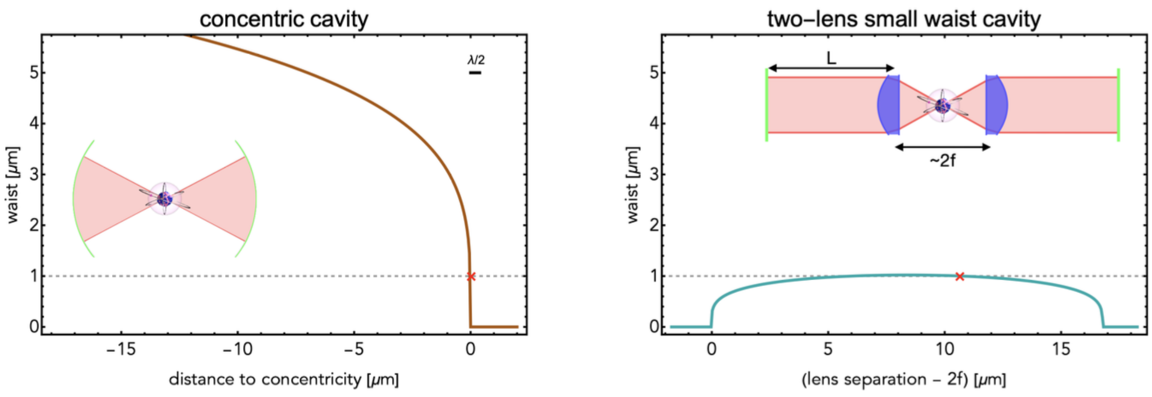

Figure S6: **Comparison between a concentric cavity and small-waist 2-lens cavity** at fixed focusing power. Zooming in on the edge of the stability diagram for a concentric cavity (left) shows the unfavorable scaling of waist size vs distance to concentricity. The scale bar indicating $\frac{\lambda}{2}$ reflects that cavity modes are actually discretely spaced by this separation, as opposed to the continuous line shown. In contrast, a two lens cavity (right) using cheap, commercially available aspheric lenses admits a broad region of stability producing a 1 μm mode waist. Varying the length L of the low-NA path between the lens and end mirror tunes the waist size at the peak of this stability region as $w_{max} = \sqrt{\bar{\lambda}\frac{f}{\frac{L}{f}-1}}$, where f is the focal length of the lens. The waist can be seen as the geometric mean of λ and the lens focal divided by length of the low-NA arm in units of f

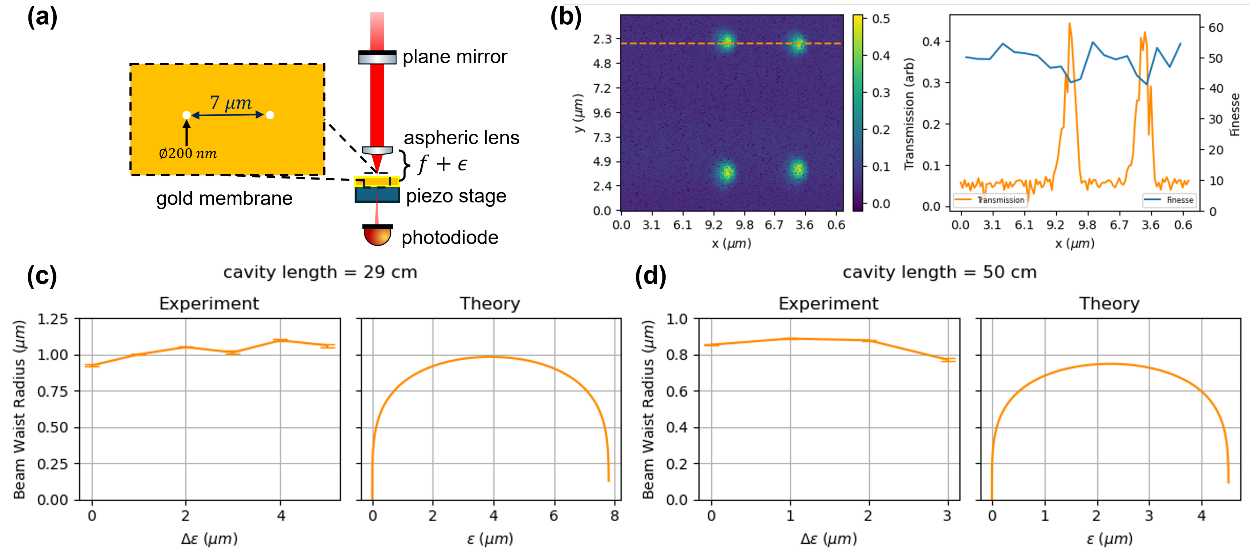

Figure S7: **Measurement of the sub-micron Mode Waist.** **(a)** Schematic of measurement setup. We built a similar cavity setup as in the main text and placed a gold membrane with pinhole with 200 nm diameter and pitch of 7 μm on a piezo scanning stage behind the aspherical lens. The extra distance ϵ is tunable via the translation stage holding the asphere. The piezo stage scans the 2D transverse plane as the transmission signal is recorded by a photodiode. **(b)** 2D Plot of transmission. As cavity mode crosses the pinhole, the finesse drops only around 15% from 52 to 42, which allows the transmission measurement to reflect the point spread function (PSF) without a substantial impact to finesse variation/impedance matching. Note that the duplication of pinhole pairs on the upper and lower regions is due to the triangle scanning waveform in the y direction of piezo stage. **(c)-(d)** Comparison of experiment and paraxial model when cavity length is 29 cm in (c) and 50 cm in (d). Note that the x axis of experiment plots $\Delta\epsilon$ refers to relative displacements in ϵ while the x axis ϵ in theory plots refers to absolute displacements. We expect the absolute ϵ values in the experiment overlap with the central parts of theory ϵ values. The scale in x direction was calibrated by using 7 μm pitch between the pinholes and we only took the gaussian fit in the x direction as our data. The error bar is the standard deviation of the average of 4 PSF features in each transmission image.

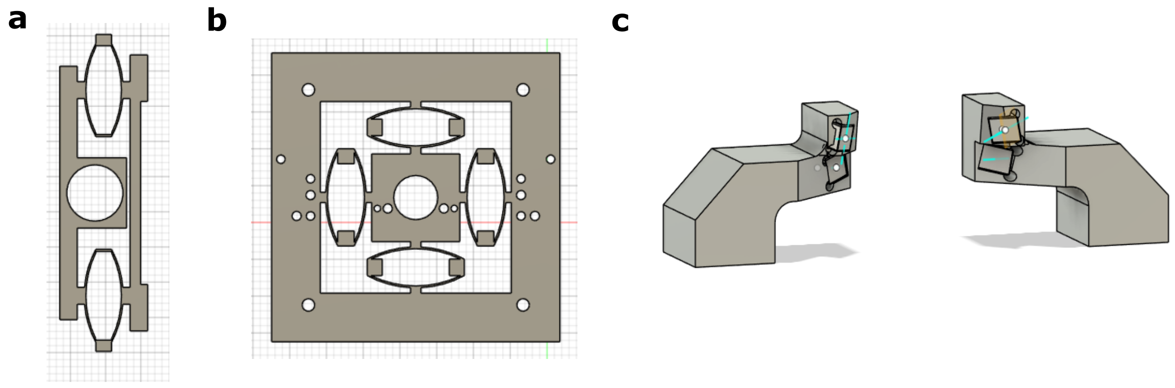

Figure S8: **Stainless steel flexures and mounts for precision alignment.** **(a)** The vertical flexure allows for control of the y-position of the curved mirror. Piezo-electric stacks slide into both holders depicted here. **(b)** 2D flexure allows for control of the x and z positions of the aspheric lens. Together with the vertical flexure, all degrees of freedom in the relative alignment between the asphere and curved mirror are accounted for. Piezo stacks slide into only half off the holders here. **(b)** Molasses mounts, with 3D angles set by proper alignment of beams to the small waist. **(c)** Molasses mounts, shaped to hold prism mirrors. The odd, 3D angles of the insets positions the beams at angles necessary for 3D molasses cooling. The mounts attach to the sides of the horizontal flexure picture in panel c.

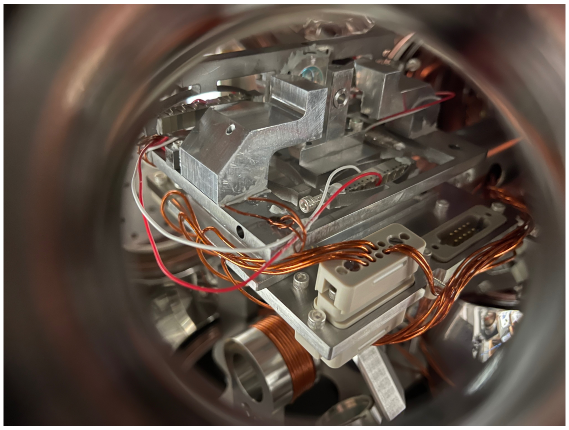

Figure S9: **The fully-assembled cavity mount under vacuum.** In vacuum installation of the completed flexure assembly, with aspheric lens and curved mirror glued into stainles steel mounts.

REFERENCES AND NOTES

1. I. Teper, Y.-J. Lin, V. Vuletić, Resonator-aided single-atom detection on a microfabricated chip. *Phys. Rev. Lett.* **97**, 023002 (2006).

2. J. Bochmann, M. Mücke, C. Guhl, S. Ritter, G. Rempe, D. L. Moehring, Lossless state detection of single neutral atoms. *Phys. Rev. Lett.* **104**, 203601 (2010).

3. T. Đorđević, P. Samutpraphoot, P. L. Ocola, H. Bernien, B. Grinkemeyer, I. Dimitrova, V. Vuletić, M. D. Lukin, Entanglement transport and a nanophotonic interface for atoms in optical tweezers. *Science* **373**, 1511–1514 (2021).

4. E. Deist, Y.-H. Lu, J. Ho, M. K. Pasha, J. Zeiher, Z. Yan, D. M. Stamper-Kurn, Mid-circuit cavity measurement in a neutral atom array. *Phys. Rev. Lett.* **129**, 203602 (2022).

5. I. D. Leroux, M. H. Schleier-Smith, V. Vuletić, Implementation of cavity squeezing of a collective atomic spin. *Phys. Rev. Lett.* **104**, 073602 (2010).

6. K. C. Cox, G. P. Greve, J. M. Weiner, J. K. Thompson, Deterministic squeezed states with collective measurements and feedback. *Phys. Rev. Lett.* **116**, 093602 (2016).

7. O. Hosten, N. J. Engelsen, R. Krishnakumar, M. A. Kasevich, Measurement noise 100 times lower than the quantum-projection limit using entangled atoms. *Nature* **529**, 505–508 (2016).

8. E. Pedrozo-Peñafiel, S. Colombo, C. Shu, A. F. Adiyatullin, Z. Li, E. Mendez, B. Braverman, A. Kawasaki, D. Akamatsu, Y. Xiao, V. Vuletić, Entanglement on an optical atomic-clock transition. *Nature* **588**, 414–418 (2020).

9. S. Ritter, C. Nölleke, C. Hahn, A. Reiserer, A. Neuzner, M. Uphoff, M. Mücke, E. Figueroa, J. Bochmann, G. Rempe, An elementary quantum network of single atoms in optical cavities. *Nature* **484**, 195–200 (2012).

10. A. Reiserer, G. Rempe, Cavity-based quantum networks with single atoms and optical photons. *Rev. Mod. Phys.* **87**, 1379–1418 (2015).

11. H. J. Kimble, The quantum internet. *Nature* **453**, 1023–1030 (2008).

12. K. M. Birnbaum, A. Boca, R. Miller, A. D. Boozer, T. E. Northup, H. J. Kimble, Photon blockade in an optical cavity with one trapped atom. *Nature* **436**, 87–90 (2005).

13. M. Steiner, H. M. Meyer, C. Deutsch, J. Reichel, M. Köhl, Single ion coupled to an optical fiber cavity. *Phys. Rev. Lett.* **110**, 043003 (2013).

14. J. Sterk, L. Luo, T. Manning, P. Maunz, C. Monroe, Photon collection from a trapped ion-cavity system. *Phys. Rev. A* **85**, 062308 (2012).

15. M. Keller, B. Lange, K. Hayasaka, W. Lange, H. Walther, Continuous generation of single photons with controlled waveform in an ion-trap cavity system. *Nature* **431**, 1075–1078 (2004).

16. M. Raha, S. Chen, C. M. Phenicie, S. Ourari, A. M. Dibos, J. D. Thompson, Optical quantum nondemolition measurement of a single rare earth ion qubit. *Nat. Commun.* **11**, 1605 (2020).

17. J. M. Kindem, A. Ruskuc, J. G. Bartholomew, J. Rochman, Y. Q. Huan, A. Faraon, Control and single-shot readout of an ion embedded in a nanophotonic cavity. *Nature* **580**, 201–204 (2020).

18. G. Calusine, A. Politi, D. D. Awschalom, Silicon carbide photonic crystal cavities with integrated color centers. *Appl. Phys. Lett.* **105**, 011123 (2014).

19. R. E. Evans, M. K. Bhaskar, D. D. Sukachev, C. T. Nguyen, A. Sipahigil, M. J. Burek, B. Machielse, G. H. Zhang, A. S. Zibrov, E. Bielejec, H. Park, M. Lončar, M. D. Lukin, Photon-mediated interactions between quantum emitters in a diamond nanocavity. *Science* **362**, 662–665 (2018).

20. J. L. Zhang, S. Sun, M. J. Burek, C. Dory, Y.-K. Tzeng, K. A. Fischer, Y. Kelaita, K. G. Lagoudakis, M. Radulaski, Z.-X. Shen, N. A. Melosh, S. Chu, M. Lončar, J. Vučković, Strongly cavity-enhanced spontaneous emission from silicon-vacancy centers in diamond. *Nano Lett.* **18**, 1360–1365 (2018).

21. D. Englund, A. Faraon, I. Fushman, N. Stoltz, P. Petroff, J. Vučković, Controlling cavity reflectivity with a single quantum dot. *Nature* **450**, 857–861 (2007).

22. D. Press, S. Götzinger, S. Reitzenstein, C. Hofmann, A. Löffler, M. Kamp, A. Forchel, Y. Yamamoto, Photon antibunching from a single quantum-dot-microcavity system in the strong coupling regime. *Phys. Rev. Lett.* **98**, 117402 (2007).

23. H. Tanji-Suzuki, I. D. Leroux, M. H. Schleier-Smith, M. Cetina, A. T. Grier, J. Simon, V. Vuletić, Interaction between atomic ensembles and optical resonators: Classical description. *Phys. Rev. A* **60**, 201–237 (2011).

24. A. S. Sørensen, K. Mølmer, Measurement induced entanglement and quantum computation with atoms in optical cavities. *Phys. Rev. Lett.* **91**, 097905 (2003).

25. M. Benito, J. R. Petta, G. Burkard, Optimized cavity-mediated dispersive two-qubit gates between spin qubits. *Phys. Rev. B* **100**, 081412 (2019).

26. C. J. Hood, H. Kimble, J. Ye, Characterization of high-finesse mirrors: Loss, phase shifts, and mode structure in an optical cavity. *Phys. Rev. A* **64**, 033804 (2001).

27. D. Hunger, T. Steinmetz, Y. Colombe, C. Deutsch, T. W. Hänsch, J. Reichel, A fiber Fabry-Pérot cavity with high finesse. *New J. Phys.* **12**, 065038 (2010).

28. N. Jin, C. A. McLemore, D. Mason, J. P. Hendrie, Y. Luo, M. L. Kelleher, P. Kharel, F. Quinlan, S. A. Diddams, P. T. Rakich, Micro-fabricated mirrors with finesse exceeding one million. *Optica* **9**, 965–970 (2022).

29. A. E. Siegman, *Lasers* (University Science Books, 1986).

30. A. Haase, B. Hessmo, J. Schmiedmayer, Detecting magnetically guided atoms with an optical cavity. *Opt. Lett.* **31**, 268–270 (2006).

31. A. Periwal, E. S. Cooper, P. Kunkel, J. F. Wienand, E. J. Davis, M. Schleier-Smith, Programmable interactions and emergent geometry in an array of atom clouds. *Nature* **600**, 630–635 (2021).

32. J. Ningyuan, A. Georgakopoulos, A. Ryou, N. Schine, A. Sommer, J. Simon, Observation and characterization of cavity Rydberg polaritons. *Phys. Rev. A* **93**, 041802 (2016).

33. L. W. Clark, N. Schine, C. Baum, N. Jia, J. Simon, Observation of Laughlin states made of light. *Nature* **582**, 41–45 (2020).

34. Y.-T. Chen, M. Szurek, B. Hu, J. de Hond, B. Braverman, V. Vuletic, High finesse bow-tie cavity for strong atom-photon coupling in Rydberg arrays. *Opt. Express* **30**, 37426–37435 (2022).

35. T. Juffmann, B. B. Klopfer, T. L. Frankort, P. Haslinger, M. A. Kasevich, Multi-pass microscopy. *Nat. Commun.* **7**, 12858 (2016).

36. P. L. Ocola, I. Dimitrova, B. Grinkemeyer, E. Guardado-Sanchez, T. Đorđević, P. Samutpraphoot, V. Vuletić, M. D. Lukin, Control and entanglement of individual rydberg atoms near a nanoscale device. *Phys. Rev. Lett.* **132**, 113601 (2024).

37. M. Jaffe, L. Palm, C. Baum, L. Taneja, J. Simon, Aberrated optical cavities *Phys. Rev. A* **104**, 013524 (2021).

38. C. Yin, H. Ando, M. Stone, D. Shadmany, A. Soper, M. Jaffe, A. Kumar, J. Simon, A cavity loadlock apparatus for next-generation quantum optics experiments. *Rev. Sci. Instrum.* **94**, 083202 (2023).

39. B. Ueberholz, S. Kuhr, D. Frese, V. Gomer, D. Meschede, Cold collisions in a high-gradient magneto-optical trap. *J. Phys. B At. Mol. Opt. Phys.* **35**, 4899–4914 (2002).

40. N. Schlosser, G. Reymond, I. Protsenko, P. Grangier, Sub-poissonian loading of single atoms in a microscopic dipole trap. *Nature* **411**, 1024–1027 (2001).

41. G. Hechenblaikner, M. Gangl, P. Horak, H. Ritsch, Cooling an atom in a weakly driven high-cavity. *Phys. Rev. A* **58**, 3030 (1998).

42. M. Norcia, A. Young, A. Kaufman, Microscopic control and detection of ultracold strontium in optical-tweezer arrays. *Phys. Rev. X* **8**, 041054 (2018).

43. H. J. Manetsch, G. Nomura, E. Bataille, K. H. Leung, X. Lv, M. Endres, A tweezer array with 6100 highly coherent atomic qubits. arXiv:2403.12021 [quant-ph] (2024).

44. D. Bluvstein, S. J. Evered, A. A. Geim, S. H. Li, H. Zhou, T. Manovitz, S. Ebadi, M. Cain, M. Kalinowski, D. Hangleiter, J. P. Bonilla Ataides, N. Maskara, I. Cong, X. Gao, P. Sales Rodriguez, T. Karolyshyn, G. Semeghini, M. J. Gullans, M. Greiner, V. Vuletić, M. D. Lukin, Logical quantum processor based on reconfigurable atom arrays. *Nature* **626**, 58–65 (2024).

45. A. Fuhrmanek, R. Bourgain, Y. R. P. Sortais, A. Browaeys, Light-assisted collisions between a few cold atoms in a microscopic dipole trap. *Phys. Rev. A* **85**, 062708 (2012).

46. Y. Chen, Y. Huang, Y. Lin, Y.-F. Chen, Intracavity PPLN crystals for ultra-low-voltage laser Q-switching and high-efficiency wavelength conversion. *Appl. Phys. B* **80**, 889–896 (2005).

47. D. D. Hudson, K. W. Holman, R. J. Jones, S. T. Cundiff, J. Ye, D. J. Jones, Mode-locked fiber laser frequency-controlled with an intracavity electro-optic modulator. *Opt. Lett.* **30**, 2948–2950 (2005).

48. I.-C. Benea-Chelmus, M. L. Meretska, D. L. Elder, M. Tamagnone, L. R. Dalton, F. Capasso, Electro-optic spatial light modulator from an engineered organic layer. *Nat. Commun.* **12**, 5928 (2021).

49. M. Endres, H. Bernien, A. Keesling, H. Levine, E. R. Anschuetz, A. Krajenbrink, C. Senko, V. Vuletic, M. Greiner, M. D. Lukin, Atom-by-atom assembly of defect-free one-dimensional cold atom arrays. *Science* **354**, 1024–1027 (2016).

50. D. Barredo, S. De Léséleuc, V. Lienhard, T. Lahaye, A. Browaeys, An atom-by-atom assembler of defect-free arbitrary two-dimensional atomic arrays. *Science* **354**, 1021–1023 (2016).

51. K. C. Cox, G. P. Greve, B. Wu, J. K. Thompson, Spatially homogeneous entanglement for matter-wave interferometry created with time-averaged measurements. *Phys. Rev. A* **94**, 061601 (2016).

52. J. McKeever, *Trapped atoms in cavity QED for quantum optics and quantum information* (California Institute of Technology, 2004).

53. M. Adamič, A. Trost, A fast high-resolution time-to-digital converter implemented in a Zynq 7010 SoC, in *2019 Austrochip Workshop on Microelectronics (Austrochip)* (IEEE, 2019), pp. 29–34.

54. J. Simon, *Cavity QED With Atomic Ensembles* (Harvard University, 2010).

55. T. Pellizzari, S. A. Gardiner, J. I. Cirac, P. Zoller, Decoherence, continuous observation, and quantum computing: A cavity QED model. *Phys. Rev. Lett.* **75**, 3788–3791 (1995).

56. G.-W. Lin, X.-B. Zou, M.-Y. Ye, X.-M. Lin, G.-C. Guo, Quantum SWAP gate in an optical cavity with an atomic cloud. *Phys. Rev. A* **77**, 064301 (2008).

57. M. C. Wang, G. E. Uhlenbeck, On the theory of the Brownian motion II. *Rev. Mod. Phys.* **17**, 323–342 (1945).

58. P. C. Chaumet, Fully vectorial highly nonparaxial beam close to the waist. *J. Opt. Soc. Am. A Opt. Image Sci. Vis.* **23**, 3197–3202 (2006).

59. F. Ferri, S. Garcia, M. Baghdad, J. Reichel, R. Long, Mapping optical standing-waves of an open-access Fabry-Pérot cavity with a tapered fiber. *Rev. Sci. Instrum.* **91**, 033104 (2020).

60. H. A. Bethe, Theory of diffraction by small holes. *Phys. Rev.* **66**, 163 (1944).

61. I. S. Madjarov, *Entangling, Controlling, and Detecting Individual Strontium Atoms in Optical Tweezer Arrays* (California Institute of Technology, 2021).
